# Spatio-temporal Remodeling of Functional Membrane Microdomains Organizes the Signaling Networks of a Bacterium

**DOI:** 10.1371/journal.pgen.1005140

**Published:** 2015-04-24

**Authors:** Johannes Schneider, Teresa Klein, Benjamin Mielich-Süss, Gudrun Koch, Christian Franke, Oscar P. Kuipers, Ákos T. Kovács, Markus Sauer, Daniel Lopez

**Affiliations:** 1 Research Center for Infectious Diseases ZINF, University of Würzburg, Würzburg, Germany; 2 Department of Biotechnology and Biophysics, University of Würzburg, Würzburg, Germany; 3 Molecular Genetics Group,Groningen Biomolecular Sciences and Biotechnology Institute, University of Groningen, Groningen, The Netherlands; 4 Terrestrial Biofilms Group, Institute of Microbiology, Friedrich Schiller University of Jena, Jena, Germany; Universidad de Sevilla, Spain

## Abstract

Lipid rafts are membrane microdomains specialized in the regulation of numerous cellular processes related to membrane organization, as diverse as signal transduction, protein sorting, membrane trafficking or pathogen invasion. It has been proposed that this functional diversity would require a heterogeneous population of raft domains with varying compositions. However, a mechanism for such diversification is not known. We recently discovered that bacterial membranes organize their signal transduction pathways in functional membrane microdomains (FMMs) that are structurally and functionally similar to the eukaryotic lipid rafts. In this report, we took advantage of the tractability of the prokaryotic model *Bacillus subtilis* to provide evidence for the coexistence of two distinct families of FMMs in bacterial membranes, displaying a distinctive distribution of proteins specialized in different biological processes. One family of microdomains harbors the scaffolding flotillin protein FloA that selectively tethers proteins specialized in regulating cell envelope turnover and primary metabolism. A second population of microdomains containing the two scaffolding flotillins, FloA and FloT, arises exclusively at later stages of cell growth and specializes in adaptation of cells to stationary phase. Importantly, the diversification of membrane microdomains does not occur arbitrarily. We discovered that bacterial cells control the spatio-temporal remodeling of microdomains by restricting the activation of FloT expression to stationary phase. This regulation ensures a sequential assembly of functionally specialized membrane microdomains to strategically organize signaling networks at the right time during the lifespan of a bacterium.

## Author Summary

Cellular membranes organize proteins related to signal transduction, protein sorting and membrane trafficking into the so-called lipid rafts. It has been proposed that the functional diversity of lipid rafts would require a heterogeneous population of raft domains with varying compositions. However, a mechanism for such diversification is not known due in part to the complexity that entails the manipulation of eukaryotic cells. The recent discovery that bacteria organize many cellular processes in membrane microdomains (FMMs), functionally similar to the eukaryotic lipid rafts, prompted us to explore FMMs diversity in the bacterial model *Bacillus subtilis*. We show that diversification of FMMs occurs in cells and gives rise to functionally distinct microdomains, which compartmentalize distinct signal transduction pathways and regulate the expression of different genetic programs. We discovered that FMMs diversification does not occur randomly. Cells sequentially regulate the specialization of the FMMs during cell growth to ensure an effective and diverse activation of signaling processes.

## Introduction

Cells typically compartmentalize their cellular processes into subcellular structures (e.g. organelles) to optimize their efficiency and improve their activity. One of the most interesting concepts in cellular compartmentalization is the proposed existence of *lipid rafts* in the membranes of eukaryotic cells [1]. Eukaryotic membranes organize a large number of proteins related to signal transduction, protein sorting and membrane trafficking into discrete nano-scale domains termed lipid rafts [1,2]. The functional diversity of lipid rafts is currently attributed to a different lipid and protein composition, as compelling evidence suggests that a heterogeneous population of lipid rafts could exist on a given cell [3–5]. Yet, the molecular mechanisms by which cells generate and regulate raft heterogeneity are still unclear. In eukaryotic systems, it is known that the integrity of lipid rafts requires the activity of two different raft-associated proteins termed flotillins (FLO-1 and FLO-2) [6,7]. Flotillins are scaffolding proteins, which may redundantly act as chaperones in recruiting the protein cargo to lipid rafts and interact with the recruited proteins that activate the signal transduction processes [8–10]. Consequently, the perturbation of the activity of flotillins causes serious defects in several signal transduction and membrane trafficking processes, which seems to be intimately related to the occurrence of severe human diseases, such as Alzheimer’s disease, Parkinson’s disease or muscular dystrophy (reviewed in [11]).

The spatial organization of signaling networks in lipid rafts has been considered a hallmark in cellular complexity because their existence is exclusively associated with eukaryotic cells. However, we recently discovered that bacteria organize many proteins related to signal transduction in functional membrane microdomains (FMMs) that are structurally and functionally similar to the lipid rafts of eukaryotic cells [12]. Bacterial flotillins are important components for the organization and the maintenance of the architecture of FMMs. Similar to the eukaryotic flotillins, bacterial flotillins probably act as scaffolding proteins in tethering protein components to the FMMs, thereby facilitating their efficient interaction and oligomerization and to mediate the efficient activation of signal transduction pathways harbored in FMMs. Consequently, mutants lacking flotillins show a severe defect in FMM-localized signaling pathways concomitantly with a severe dysfunction of diverse physiological processes, such as biofilm formation, natural competence or sporulation [12–17].

The FMMs of the bacterial model *Bacillus subtilis* contain two different flotillin-like proteins, FloA and FloT [12]. FloA and FloT flotillins physically interact [13] and presumably play a redundant role because the dysfunction of specific FMM-associated physiological processes, like biofilm formation, only occurs in the Δ*floA* Δ*floT* defective mutant and is not observed in either of the Δ*floA* or Δ*floT* single mutants [17]. Likewise, the overexpression of both *floA* and *floT* causes pleiotropic effects in cell division and cell differentiation but this effect is not observed in cells that overexpress one single flotillin gene [16]. In this respect, bacterial flotillins seem to behave similarly to human flotillins FLO-1 and FLO-2, given that both FLO-1 and FLO-2 are associated with each other in hetero-oligomeric complexes and have a strong regulatory correlation [18–20]. These experimental evidences led to the general assumption that both flotillins play a redundant function in both eukaryotic lipid rafts and bacterial FMMs.

In this report, we provide evidence that a heterogeneous population of membrane microdomains coexists on bacterial cells. We show that FloA and FloT are two functionally different flotillins that physically interact but unevenly distribute within the FMMs of bacterial cells. FloA and FloT act as specific scaffold proteins that tether a defined group of FMMs-associated proteins. This generates functionally distinct microdomains, which compartmentalize distinct signal transduction pathways and regulate different genetic programs. Importantly, we show that cells sequentially regulate the functional specialization of the FMMs during cell growth. Cells restrict the expression of the *floT* gene to stationary phase to ensure an effective activation of signaling processes at specific times during the lifespan of the bacterium.

## Results

### FloA and FloT are differentially regulated in *B*. *subtilis*

While exploring flotillin redundancy in the FMMs of *B*. *subtilis*, we discovered that the expression of FloA and FloT is controlled by different genetic programs, which could indicate that these are two functionally different flotillins. We came across this finding by examining the expression profiles of *floA* and *floT* genes in the 249 different growing conditions that are published in [21,22], and are available in SubtiExpress (http://subtiwiki.uni-goettingen.de/apps/expression). By doing this, we consistently found high expression of *floA* in all the growing conditions tested, including LB and MSgg growth media, the two growth media that we normally used in the laboratory to grow *B*. *subtilis* (S1A Fig) [23]. However, the expression of *floT* showed more variability among the growth conditions tested and exhibited an important difference in gene expression between LB (lower expression of *floT*) and MSgg (higher expression of *floT*). To test this in the laboratory, we constructed *B*. *subtilis* strains harboring the P_*floA*_-*yfp* and P_*floT*_-*yfp* transcriptional fusions (YFP is yellow fluorescence protein) and grew them in LB and MSgg media [23]. Our laboratory uses the chemically-defined medium MSgg to induce sporulation and the formation of robust biofilms in *B*. *subtilis* cultures and differs to LB medium in which *B*. *subtilis* did not show any of the developmental characteristics of MSgg [24]. By growing *B*. *subtilis* cells in these two growth conditions, we detected an activation of *floT* expression in MSgg (S1A Fig), while LB medium showed poor activation of *floT* expression (S1B Fig). In contrast, *floA* was equally expressed in both MSgg and LB media. Furthermore, we generated strains labeled with the FloA-GFP and FloT-GFP translational fusions (GFP is green fluorescence protein) to visualize and quantify flotillin protein production using flow cytometry. The FloT-GFP labeled strain showed a reduction of the fluorescence signal when grown in LB medium while FloA-GFP was equally expressed in both MSgg and LB media (S1C Fig).

**Fig 1:**
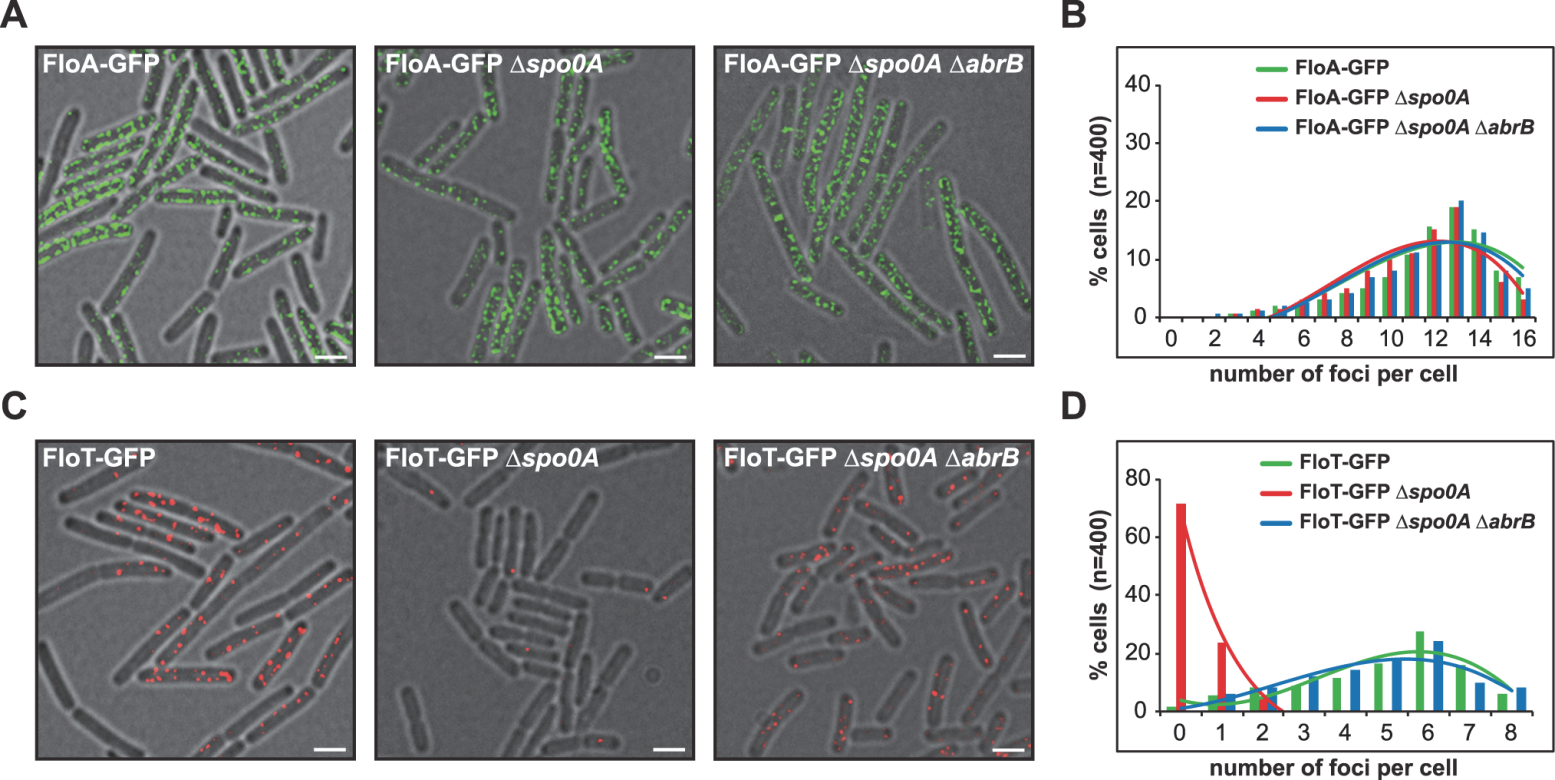
Spo0A regulates *floT* and not *floA* expression via inhibition of AbrB. **(A)** Fluorescence microscopy pictures of different strains expressing FloA-GFP translational fusion (Fluorescence signal in green). Scale bars are 2 μm. **(B)** Quantification of fluorescence foci of different strains expressing FloA-GFP translational fusion. **(C)** Fluorescence microscopy pictures of different strains expressing FloT-GFP translational fusion (Fluorescence signal in red). Scale bars are 2 μm. **(D)** Quantification of fluorescence foci of different strains expressing FloT-GFP translational fusion. Cells were grown in MSgg medium at 37°C until stationary phase.

To investigate whether the differential production of FloA and FloT is a cell-regulated process, the strains labeled with P_*floA*_-*yfp* and P_*floT*_-*yfp* transcriptional fusions were used to systematically inactivate regulatory genes of *B*. *subtilis* and search for mutants capable of altering the expression of *floT* in MSgg medium (S1D Fig). We detected a uniform expression of *floA* in all mutants tested. However, we discovered that cells lacking the *abrB* gene showed increased expression of *floT*. Additionally, we found inhibition of *floT* expression in cells when the *spo0A* gene was deleted. Importantly, *spo0A* and *abrB* belong to the same signaling pathway. AbrB is a repressor of biofilm formation among other processes [25] and its expression is negatively regulated by Spo0A [26]. Spo0A is a master regulatory protein necessary for the activation of many physiological processes related to stationary phase [27]. Therefore, this provides epistatic evidence that Spo0A positively regulates *floT* expression at stationary phase via inhibition of *abrB* and that this genetic cascade does not affect the expression of *floA*. To test this hypothesis, we deleted *spo0A* and/or *abrB* genes in FloA-GFP and FloT-GFP labeled strains and monitored the subcellular distribution pattern of flotillins using fluorescence microscopy and applying a deconvolution algorithm to eliminate out-of-focus signal and to improve their correct visualization (see material and methods section) (Fig 1). Indeed, Δ*spo0A* or Δ*spo0A* Δ*abrB* mutants showed no variation in the distribution pattern of fluorescence foci that were generated by FloA (Fig 1A and 1B). In contrast, Δ*spo0A* mutant showed a severe reduction of fluorescence foci that were generated by FloT, which could be reconstituted in the Δ*spo0A* Δ*abrB* double mutant (Fig 1C and 1D).

Activation of Spo0A (Spo0A~P) occurs at stationary phase due in part to the activation of the histidine kinase C (KinC) [28,29], which is driven by the action of the self-produced signaling molecule surfactin. Thus, FloA-GFP and FloT-GFP labeled strains were grown in LB medium and complemented with exogenously added surfactin (5 μM) (S2A and S2C Fig). FloA-GFP labeled cells showed no alteration of the fluorescence signal but FloT-GFP labeled cells showed an increase in the number of foci (S2A–S2D Fig). This is a Spo0A-depedent effect because the *spo0A* deficient strain showed no recovery of FloT expression upon addition of surfactin (S2E and S2F Fig). Altogether, these results show an upregulation of FloT production at stationary phase in a Spo0A-dependent manner likely via AbrB. In contrast, the production of FloA is not influenced by this regulatory cascade.

### Flotillins distribute unevenly within the FMMs of *B*. *subtilis*

**Fig 2:**
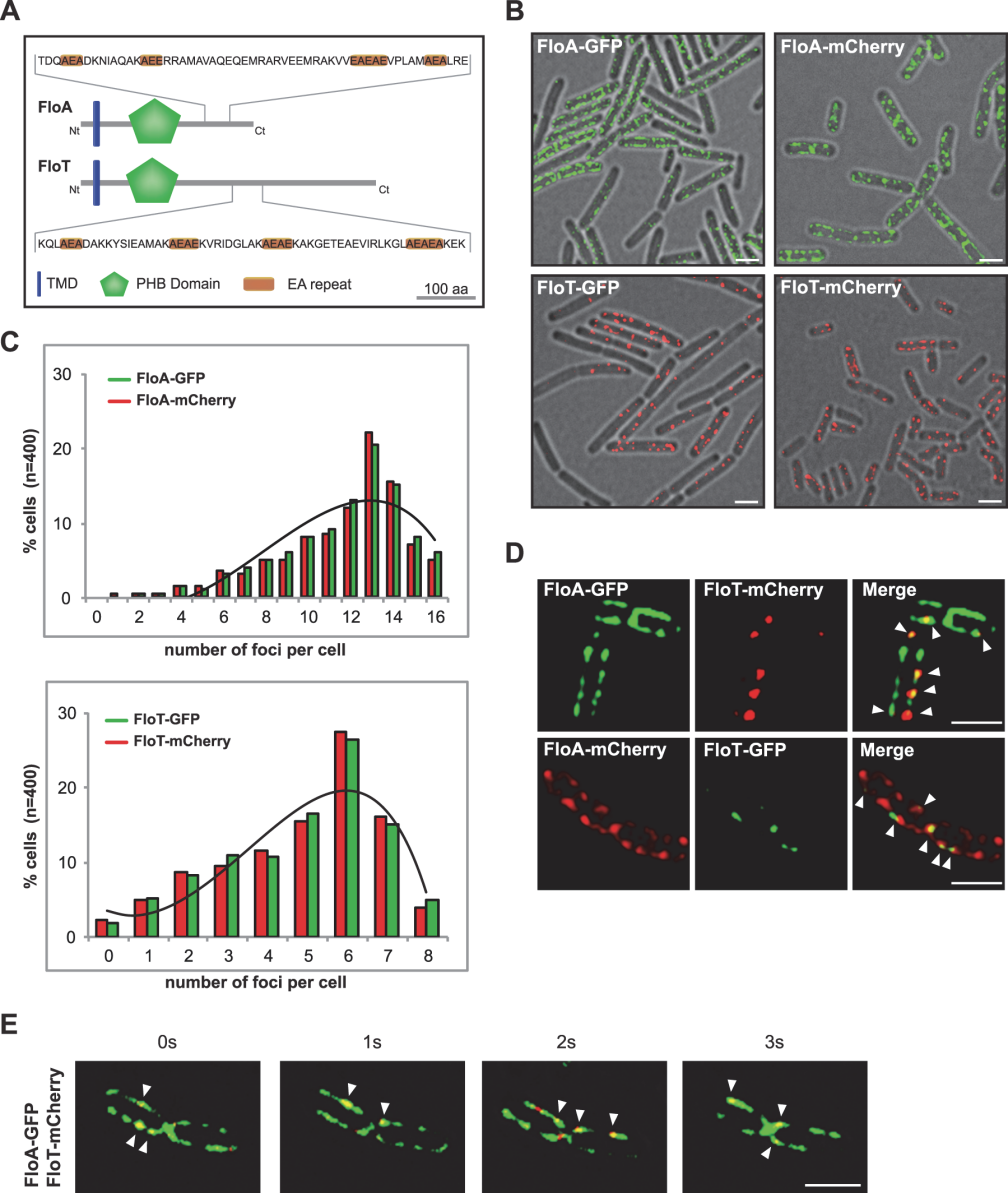
FloA and FloT are two distinct flotillins. **(A)** Comparative diagram of FloA and FloT protein structures. The membrane-anchored region is represented in blue. The PHB domain is represented in green and the coil-coiled region is magnified and EA repeats are labeled in orange. Scale bar is 100 amino acids. **(B)** Fluorescence microscopy pictures of cells labeled with FloA-GFP, FloA-mCherry (upper panel), FloT-GFP and FloT-mCherry (lower panel) translational fusions. Fluorescence signal associated with FloA is represented in green and fluorescence signal associated with FloT is represented in red. Cultures were grown in MSgg medium at 37°C until stationary phase. Scale bars are 2 μm. **(C)** Quantification of the number of foci per cell (n = 400). **(D)** Fluorescence microscopy pictures of double-labeled strains. Cultures were grown in MSgg medium at 37°C until stationary phase. GFP signal is represented in green and mCherry signal is represented in red. Right panel shows the merge of the two fluorescence signals, which is visualized as yellow fluorescence signal. Scale bars are 2 μm. **(E)** Time lapse fluorescence microscopy analysis of cells expressing FloA-GFP (green signal) and FloT-mCherry (red signal) translational fusions. Signal was monitored within the same cells at 1 sec intervals. Cultures were grown in MSgg medium at 37°C until stationary phase. Scale bar is 2 μm.

The distinct regulatory programs for FloA and FloT production led us to hypothesize that FloA and FloT may play different roles in *B*. *subtilis* cells. To investigate this hypothesis, we first explored whether FloA and FloT show any structural difference. FloT is a larger protein (509 aa) that has an extended C-terminal region compared to FloA (331 aa) (Figs 2A and S3A). To determine if these structural differences are associated with a different subcellular distribution pattern, we used strains labeled with the FloA-GFP and FloT-GFP translational fusions to visualize and quantify the number of fluorescent foci (n = 400) using fluorescence microscopy. On average, FloA distributed in 13 foci per cell while FloT distributed approximately in 6 foci (Figs 2B, 2C and S3B). These results are consistent with the number of foci that we detected in the genetic analysis that are shown in Fig 1. However, to validate that these results were not a consequence of clustering artifacts [30], we compared their distribution pattern using non-dimerizing monomeric red fluorescence protein mCherry (mCh) in a total of 400 cells. Likewise, FloA distributed in 13 foci per cell while FloT distributed in 6 foci, as previously observed (Figs 2B, 2C and S3B). Importantly, the subcellular localization of flotillins consistently showed that FloA distributed in more foci per cell than FloT. To gain more insight about the differential distribution pattern of flotillins, we performed co-localization experiments using FloA-GFP, FloT-mCh and FloA-mCh, FloT-GFP double-labeled strains. Co-localization of both signals was detected by fluorescence microscopy, showing colocalization of FloT with FloA in all cells examined (Fig 2D), which adds to the previous notion that FloA and FloT physically interact [12,13,17]. However, the obvious differences in the number of foci between FloA and FloT resulted in the colocalization of both FloA and FloT signals only in a subset of foci (Pearson’s correlation coefficient R^2^ = 0.81). This diversified the pool of FMMs of a given cell into two different types of microdomains: one family of microdomains that contains solely FloA signal and a second type of microdomains in which both FloA and FloT signals converge. We performed time-lapse fluorescence microscopy experiments using a FloA-GFP FloT-mCherry double-labeled strain to investigate the dynamics of the subcellular co-localization. A series of images were taken at one-second time interval (Fig 2E). The fluorescence signal attributable to FloA and FloT reorganized dynamically within the membrane and consistently showed co-localization of signals from FloA and FloT.

**Fig 3:**
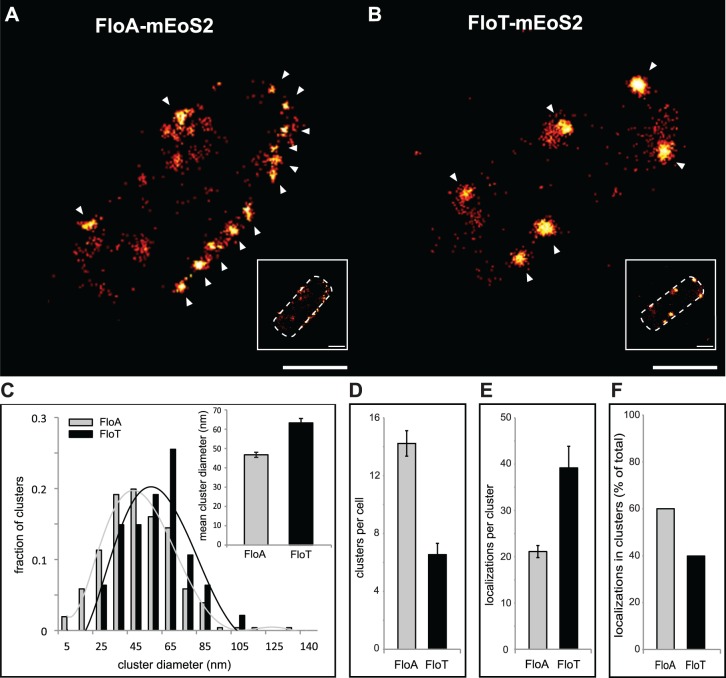
FloA and FloT distribute differently within the bacterial microdomains. **(A and B)** PALM images of cells labeled with FloA-mEoS2 (A) or FloT-mEoS2 (B) translational fusions grown to stationary phase and fixed with PFA (4%). With increasing localization density the color code changes from red to yellow. White arrows indicate the localization of a cluster. Scale bars are 500 nm. Detail of the right bottom of each panel shows a dashed-line decorated PALM picture as a general indicator of the cell outline. Scale bar is 500 nm. **(C)** Comparative graph of the diameter of the clusters that were generated by FloA-mEoS2 and FloT-mEoS2 fluorescence signal. The upper right corner shows a graph with the mean of the diameter of the FloA and FloT clusters. **(D)** Comparative graph of the number of clusters detected in FloA-mEoS2 and FloT-mEoS2 labeled cells. **(E)** Comparative graph of the number of FloA and FloT localizations per cluster. **(F)** Comparative graph of the percentage of localizations that organized in clusters.

The asymmetrical distribution pattern of FloA and FloT was further examined at higher resolution using super-resolution imaging by PALM [31,32]. To this end, FloA and FloT were labeled with the photoactivatable monomeric protein mEOS2 and expressed in *B*. *subtilis* cells. FloA-mEOS2 and FloT-mEOS2 proteins were activated by low intensity irradiation at 405 nm. Photoactivated proteins were excited at 568 nm, imaged and bleached before the next cycle of photoactivation. Individual protein positions were determined (localized) in each image frame and used to reconstruct a high-resolution PALM image (Fig 3A and 3B). Clusters candidates were defined by either one connected pixel area in image-based analysis or by a cloud of scattered localizations with spatial coherence in localization based analysis. Spatial coherence implies that the increase local density of localizations follows a Gaussian distribution within the cluster, which is indicative of the nonrandom distribution of localizations. Using the raw localization data and the corresponding super-resolved image, we generated a mask to define possible cluster candidates and separate them from the localization pseudo background. By using this technique, we confirmed that FloA assembled in 13 small clusters per cell (Diameter = 46.73 ± 1.35 nm). FloT however, assembled in approximately 6 larger clusters per cell (Diameter = 63.39 ± 2.28 nm) with a higher content of proteins (Fig 3C–3F). To validate these results, we also monitored the distribution pattern of FloA and FloT when fused to photoactivatable monomeric PAmCherry using PALM (S4A and S4B Fig). The statistical analysis of the signals detected by PALM and further validation by western blot analysis suggested that FloT is more abundant than FloA in cells and yet, based on our results, is concentrated in a lower number of foci (S4C and S4D Fig).

### Spatial organization of flotillin distribution is driven by flotillin interaction

**Fig 4:**
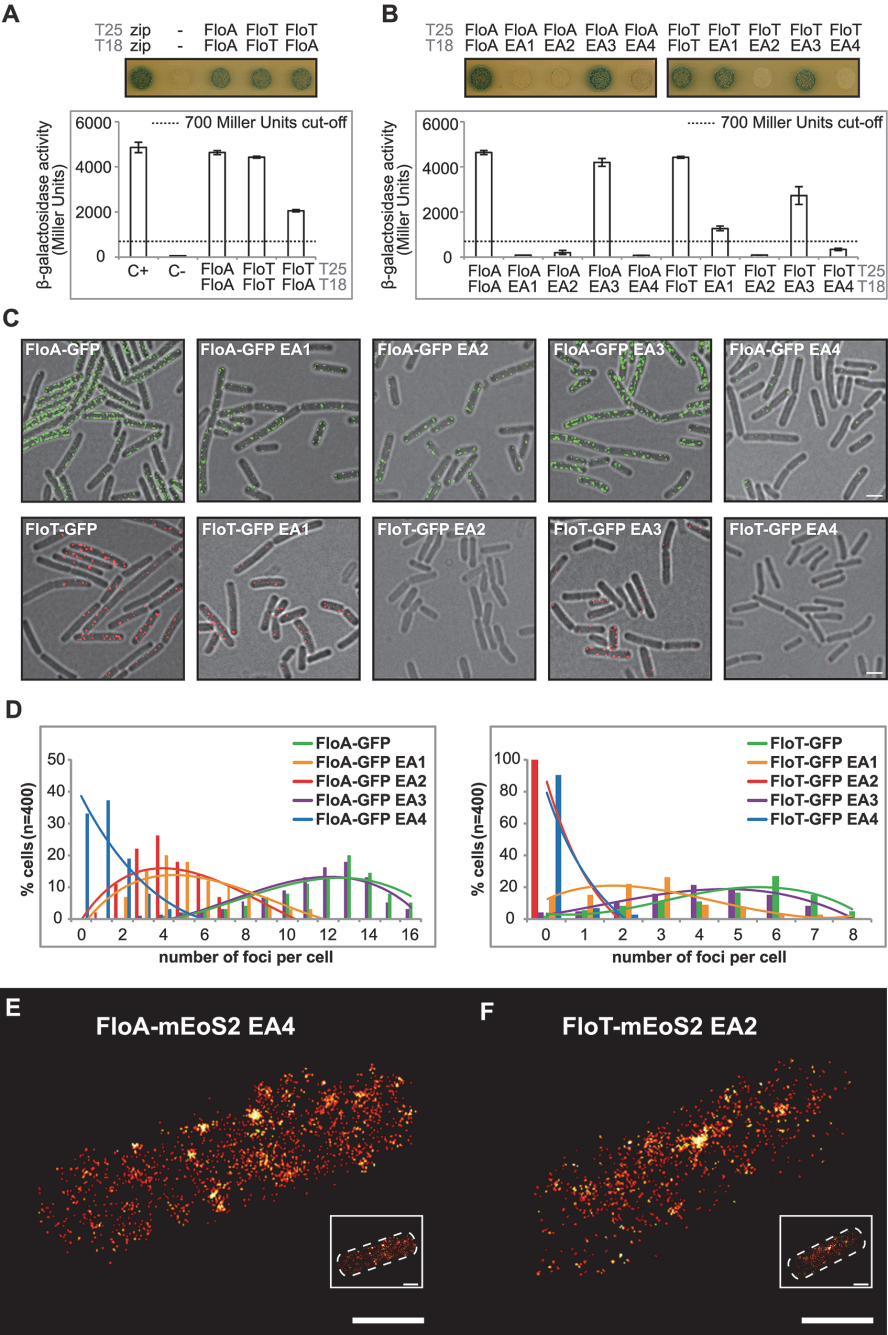
Oligomerization properties of FloA and FloT. **(A)** BTH analysis to study the interactions between FloA and FloT. Interaction activates *lacZ* and this degrades X-Gal (blue). The two cytoplasmic domains of a leucine-zipper represent a positive control (pKT25-zip + pUT18C-zip). The negative control is represented by the *E*. *coli* strains harboring empty plasmids (pKNT25 + pUT18). Plasmids containing FloA, FloT and EA variants are pKNT25 (T25) or pUT18 (T18). Dashed line indicates the threshold limit of 700 Miller Units that defines a positive (≥ 700 Miller Units) and a negative interaction signal (≤ 700 Miller Units) according to the instructions of the manufacturer. **(B)** BTH analysis between the EA1 to EA4 variants of FloA and FloT. Positive controls are wild type FloA and FloT. Dashed line indicates the threshold limit of 700 Miller Units that defines a positive and a negative interaction signal. **(C)** Fluorescence microscopy of cells expressing GFP-tagged versions of EA variants of FloA (Upper row). Fluorescence microscopy of cells expressing GFP-tagged versions of EA variants of FloT (Bottom row). Scale bar is 2 μm. (D) Quantification of the number of foci per cell (n = 400) of the distinct GFP-tagged versions of EA variants of FloA (left panel) and FloT (right panel). **(E and F)** PALM images of cells expressing the EA4 variant of FloA-mEoS2 (E) and EA2 variant of FloT-mEoS2 (F). Scale bars are 500 nm. Detail of the right bottom of each panel shows a dashed-line decorated PALM picture as a general indicator of the cell outline. Scale bar is 500 nm.

The molecular basis of the asymmetrical distribution of FloA and FloT was explored by monitoring the intra- and inter-specific interactions that occur between FloA and FloT flotillins. To do this, we used a bacterial two-hybrid (BTH) assay, in which FloA and FloT were tagged to T25 or T18 catalytic domains of an adenylate cyclase that reconstitute the enzyme upon interaction of two proteins [33]. A fully active adenylate cyclase produces cAMP, which accumulates in the cytoplasm and triggers the expression of a cAMP-inducible *lacZ* reporter gene [33]. Using this assay, we detected a strong interaction signal with FloA alone (Fig 4A) (Instructions of the manufacturer define a positive signal if above the threshold of 700 Miller Units [33]). Likewise, a strong interaction signal was detected with FloT (Fig 4A). This is indicative of the capacity of FloA and FloT to form homo-oligomers. However, when we assayed the interactions between FloA and FloT, the interaction signal was less prominent in comparison to the FloA-FloA and FloT-FloT interactions, suggesting that flotillins are prone to form homo-oligomers while hetero-oligomerization occurs to a lesser extent (Fig 4A). The propensity to form homo-oligomers suggests different interaction properties between FloA and FloT, which is probably a determinant in the generation of distinct subcellular distribution patterns.

Both FloA and FloT have a N-terminal region that anchors the protein to the membrane and the SPFH domain that is characteristic of this protein family (for stomatin, prohibitin, flotillin and HflK/C) [34,35]. However, the C-terminal region, which is the most variable region between FloA and FloT, contains four glutamate-alanine repeats (EA repeats) that are responsible for the oligomerization of human FLO-1 and FLO-2 (Figs 2A and S3A) [36] and are probably important in determining the interactions between FloA and FloT. We performed site-directed mutagenesis of the C-terminal region of each flotillin, which generated several variants of FloA and FloT, in which each one of the four EA repeats was replaced (EA→GL) (S5B Fig). We assayed the interaction properties of each one of the resultant variants using a BTH approach. FloA-FloA and FloT-FloT interactions did not occur when we altered the EA2 or EA4 repeats (≤ 700 Miller Units). Additionally, FloA-FloA interaction was abrogated when EA1 was mutated (≤ 700 Miller Units) while EA3 seemed to minimally affect the homo-oligomerization of both FloA and FloT (≥ 700 Miller Units) (Fig 4B). Moreover, the localization pattern of GFP-labeled variants was examined. Variants with EA2 and EA4 altered repeats showed poor aggregation and a severe decrease in the number of foci (Fig 4C and 4D). Alterations in EA1 affected severely the oligomerization of FloA while the variants with altered EA3 showed mild alterations in their distribution pattern (Fig 4C and 4D). None of the distribution patterns were appreciably altered in the absence of the alternative flotillin, suggesting that additional interaction motifs may exist to facilitate hetero-oligomerization (S5C Fig). Since the expression of the altered variants was still detected by western blot analysis (S5D Fig), it is possible that they become dispersed throughout the cellular membrane. Thus, we constructed a mEOS2-tagged version of FloA(EA4) and FloT(EA2) to study their subcellular distribution pattern using PALM microscopy. By using this approach, we detected a large number of single fluorescent proteins randomly dispersed across the cellular membrane (Fig 4E and 4F) rather than organized in foci.

**Fig 5:**
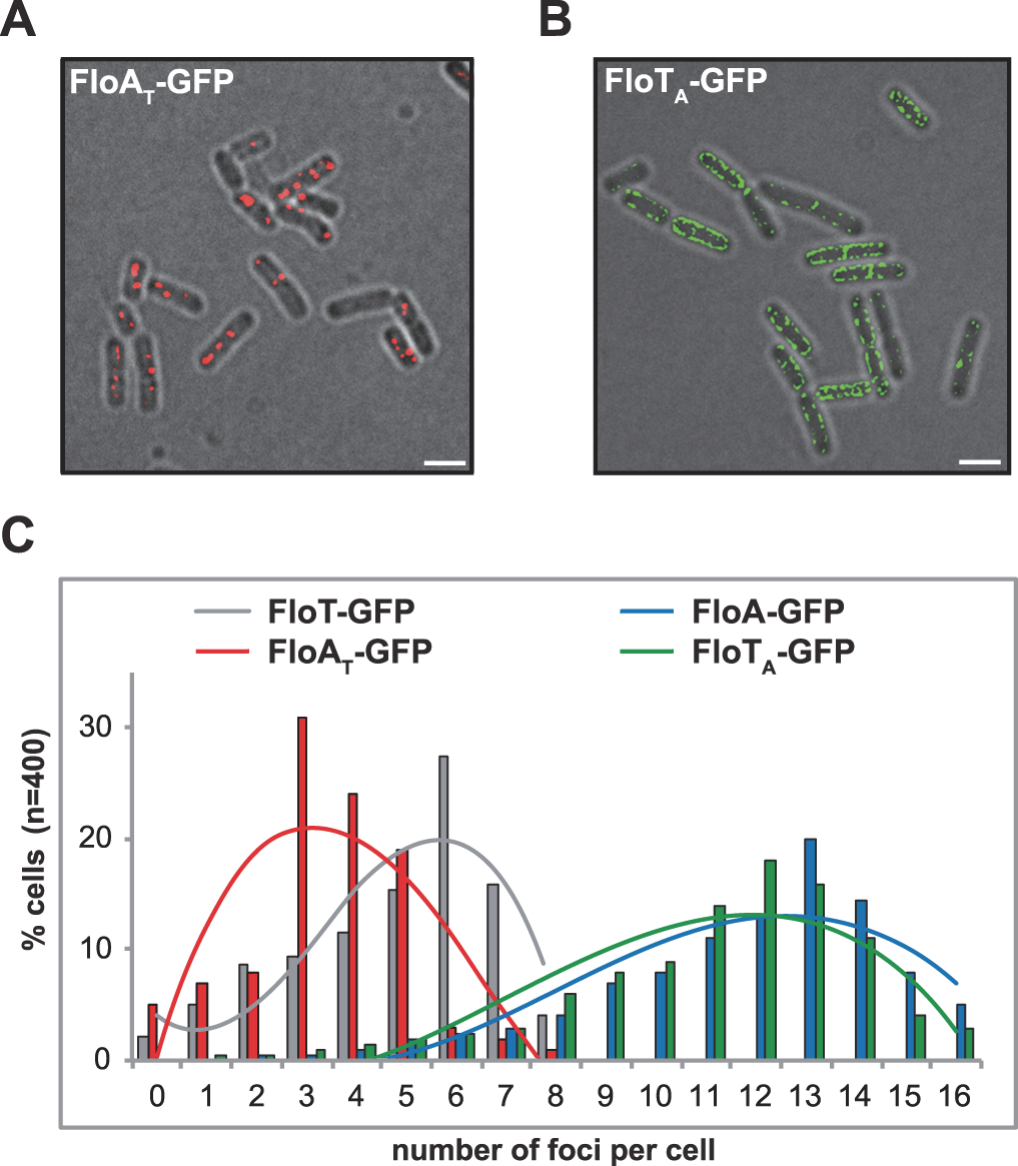
C-terminal region of FloA and FloT plays a role in their oligomerization properties. **(A)** Fluorescence microscopy image of cells expressing a GFP-tagged version of FloA_T_. Fluorescence signal is represented in red (scale bar is 2 μm) **(B)** Fluorescence microscopy image of cells expressing a GFP-tagged version of FloT_A_. Fluorescence signal is represented in green (scale bar is 2 μm). **(C)** Quantification of the number of foci per cell (n = 400) in different genetic backgrounds.

The abovementioned results suggest that FloA and FloT display distinct subcellular distribution pattern, due in part to their different oligomerization affinities, which are determined by the specific interactions that occurred at the C-terminal region of each flotillin. We confirmed these observations by generating a chimeric version of FloA that contains the C-terminal region of FloT (FloA_T_) and a chimeric version of FloT that contains the C-terminal region of FloA (FloT_A_). GFP-fused versions of these proteins were generated to examine their subcellular distribution pattern (Fig 5). Using this approach, we consistently observed that the distribution pattern of FloA_T_ resulted different from wild-type FloA and resembled the distribution pattern of wild-type FloT. Likewise, the distribution pattern of FloT_A_ resulted very different from the wild-type FloT pattern, showing approximately 13 smaller foci per cell, which is similar to the distribution of wild-type FloA. These results are in agreement with what is shown in Fig 4 and confirmed that the c-termini regions confer specific oligomerization properties to each flotillin.

### FloA and FloT tether distinct signal transduction pathways

**Fig 6:**
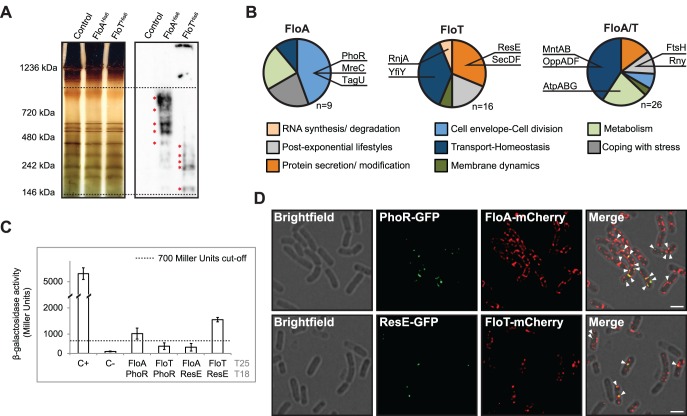
Physiological processes associated with FloA or FloT. **(A)** Silver-stained BN-PAGE that resolves the protein complexes from the membrane fraction of different strains (left panel). Western blot assay, using antibodies against His^6^, to detect flotillin-interacting protein complexes on the BN-PAGE (right panel). Dashed lines define the higher (1000 KDa) and lower (100 KDa) resolution limit of protein complexes in a 4%-20% BN-PAGE. Red asterisks denote the bands that were analyzed by MS. **(B)** Functional classification of the proteins identified in association FloA (left), FloT (centre) or both FloA and FloT (right). Dashed lines define the higher (1,000 KDa) and lower (100 KDa) resolution limit of protein complexes in a 4%-20% BN-PAGE. Red asterisks denote the bands that were analyzed by MS. **(C)** BTH assay to study the interactions between FloA or FloT and the PhoR and ResE flotillin-associated kinases. Dashed line indicates the threshold limit of 700 Miller Units that defines a positive (≥ 700 Miller Units) and a negative interaction signal (≤ 700 Miller Units) according to the instructions of the manufacturer. **(D)** Fluorescence microscopy images of colocalization of fluorescence signals. Upper row shows a double-labeled strain expressing PhoR-GFP and FloA-mCherry translational fusions. Fluorescence signals are represented in green and red, respectively. Colocalization of both green and red fluorescence signals merges in a yellow signal (indicated with a white arrow). Scale bar is 2 μm. Bottom row shows a double-labeled strain expressing ResE-GFP and FloT-mCherry translational fusions. Fluorescence signals are represented in green and red, respectively. Colocalization of both green and red fluorescence signal merges in a yellow signal (indicated with a white arrow). Scale bar is 2 μm.

We were interested in exploring the biological significance of cells expressing two different flotillins with distinct spatio-temporal distribution patterns. We hypothesized that this may occur because these are two functionally different flotillins and therefore, they serve as scaffold in tethering the components of distinct signal transduction pathways in *B*. *subtilis*. We explored this hypothesis by first identifying the proteins that distinctively bind to either FloA or FloT. To do this, His^6^-tagged versions of FloA and FloT were expressed in *B*. *subtilis* cells. The membrane fraction was resolved by blue-native PAGE (BN-PAGE) to allow the separation of the membrane protein complexes in their natural oligomeric states [37]. Our BN-PAGE assays used a polyacrylamide gradient of 4%–20%, which allows the resolution of membrane-bound protein complexes with a molecular weight between 100 kDa and 1000 kDa. BN-PAGE coupled to immunoblotting, using antibodies against the His^6^ tag, was used to identify a number of membrane-associated protein complexes that exclusively interacted with FloA or FloT (Fig 6A and S3 Table). The corresponding bands were identified by mass spectrometry (MS) and validated as components of the protein cargo of the FMMs previously identified in analyses of the DRM fraction [12,13,17] (S3 Table).

MS analysis identified nine membrane proteins exclusively associated with FloA (Fig 6B). Their functional classification suggested their active participation in processes related to cell envelope regulation and cell division regulation. Those include the cytoskeletal-associated proteins MreC and PBP1A/1B or proteins related to cell wall remodeling, such as TagU and PhoR [38] (Fig 6B). We were particularly interested in the PhoR-FloA interaction, as this is a signaling kinase that activates a cascade that is related to cell wall organization [39] and is probably representative of the contribution of FloA to the FMMs. Using a BTH assay, we confirmed a specific interaction between PhoR and FloA (≥ 700 Miller Units) that was not observed between PhoR and FloT (≤ 700 Miller Units) (Fig 6C). In contrast, a total number of sixteen proteins were identified in exclusive association with FloT and their functional classification suggested an important role in adaptation to stationary phase (Fig 6B). This is the case for YclQ, YhfQ or YfiY proteins involved in siderophore uptake (reviewed in [40]); the protein secretion components SecA, SecDF and YacD, which have been correlated to FloT in previous studies [13] and the membrane-bound sensor kinase ResE, required for antibiotic, siderophore production and adaptation to oxygen-limiting conditions [41]. BTH analysis confirmed the interaction of ResE and FloT (≥ 700 Miller Units) that was not observed between ResE and FloA (≤ 700 Miller Units) (Fig 6C). We also identified a group of twenty-six proteins that interacted with both FloA and FloT (Fig 6B). The functional classification of this group is more diverse but generally related to cell differentiation processes. This includes the metalloprotease FtsH, required for the activation of Spo0A and thus, biofilm formation and sporulation [42] and known to interact with FloA and FloT from previous studies [13,16,17] and the OppABCDF oligopeptide permease, responsible for importing peptidic signals to activate biofilm formation or natural competence [43].

**Fig 7:**
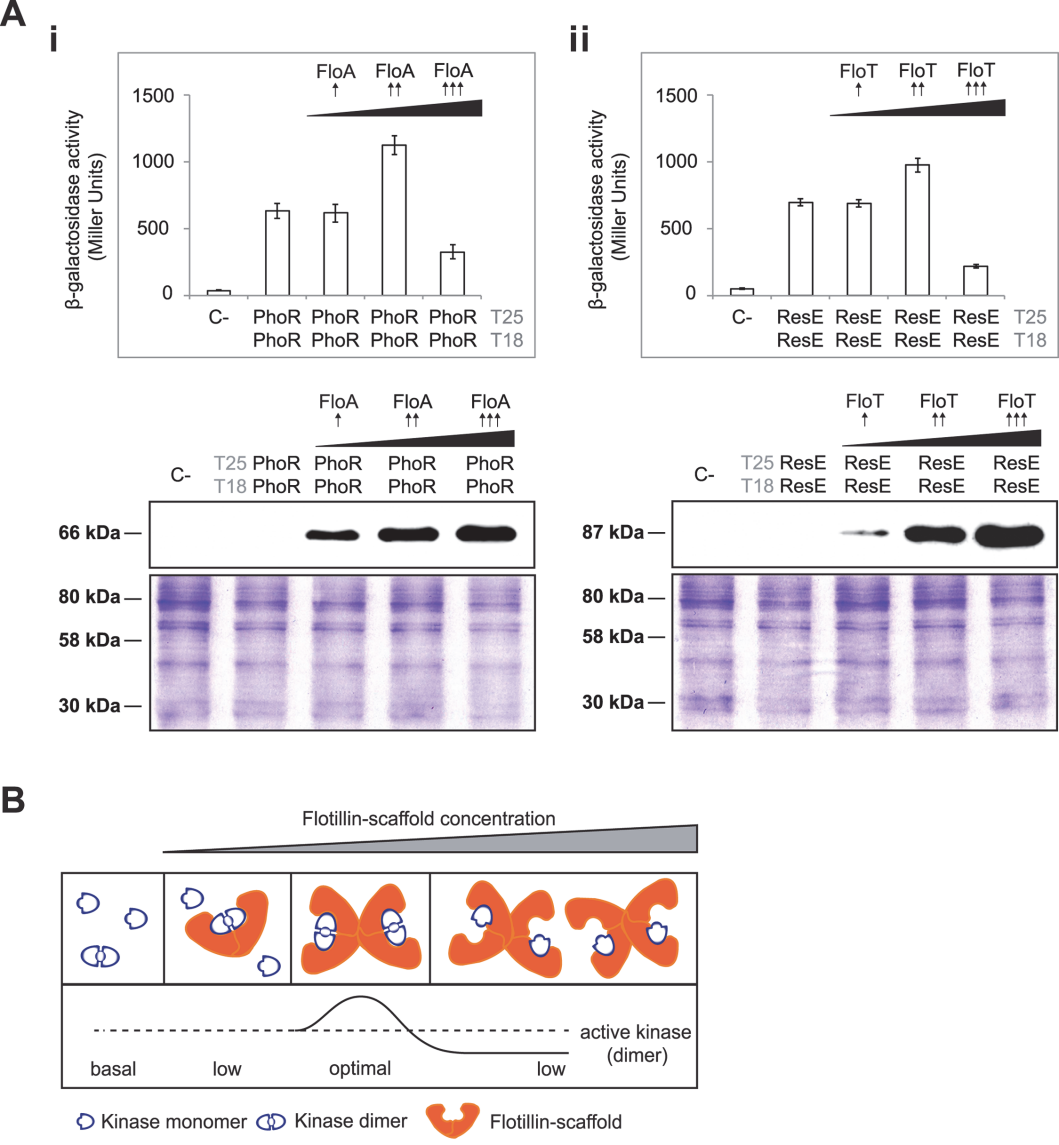
Tethering of signaling partners mediated by flotillins. **(A)** BTH assay to quantify the interaction of PhoR (i) and ResE (ii) under different concentrations of flotillins (upper panels). Dashed line indicates the threshold limit of 700 Miller Units that defines a positive (≥ 700 Miller Units) and a negative interaction signal (≤ 700 Miller Units) according to the instructions of the manufacturer. Lower- (pSEVA-621), medium- (pSEVA-631) and high-copy (pSEVA-641) plasmids expressing His^6^-tagged FloA and FloT rendered lower (⬆), medium (⬆⬆) and higher (⬆⬆⬆) concentration of flotillin in the BTH assay, respectively, according to immunoblot analysis (lower panels). SDS-PAGE are shown as loading control. **(B)** Inhibition of the activity of a protein complex by scaffold titration. Protein assembly by scaffold proteins has potential drawbacks. At high concentrations, scaffolds may titrate enzyme and substrate away from each other.

To investigate in more detail the interactions between FloA-PhoR and FloT-ResE that we discovered in the BN-PAGE and the bacterial two-hybrid analysis, we performed co-localization experiments using FloA-mCherry, PhoR-GFP and FloT-mCherry, ResE-GFP double-labeled strains. We confirmed by RT-PCR analyses that PhoR-GFP and ResE-GFP translational fusions complemented Δ*phoR* and Δ*resE* mutants respectively, which suggested that the translational fusions were functional (S6 Fig). Co-localization of FloA and PhoR signals was detected by fluorescence microscopy. Likewise, we also detected co-localization of the FloT and ResE signals (Fig 6D). Colocalization of PhoR and ResE with their respective flotillin was detected in all cells examined (Pearson’s correlation coefficients R^2^ = 0.82 and R^2^ = 0.85, respectively). These results suggest that FloA-PhoR and FloT-ResE are spatially correlated and support our hypothesis that FloA-PhoR and FloT-ResE physically interact. The specific interaction detected between PhoR and ResE sensor kinases and their respective flotillins was explored in further experiments to better understand how the scaffold activity of bacterial flotillins physically influences the activity of their signaling partners. The most direct hypothesis is that scaffold proteins facilitate signal transduction through tethering of signaling partners, because they enforce proximity and increase the likelihood of their interaction [44]. Thus, we investigated the effect of increasing concentrations of the scaffold flotillins on the interaction and activity of PhoR and ResE. PhoR and ResE belong to the PhoPR and ResDE two-component systems (TCS), which comprise a receptor histidine kinase and their cognate response regulator (PhoP and ResD). Histidine kinases are activated by forming homodimers, autophosphorylate and generate a phosphotransfer reaction to their response regulators. First, we generated a BTH assay to quantitatively monitor the homo-dimerization of PhoR and ResE (Fig 7A). This assay was complemented with a pSEVA modulable vector system [45], to generate different strains that produced lower, medium and higher levels of their respective flotillins that were further validated by immunoblotting (Fig 7A). These strains were used to quantitatively monitor the homo-dimerization efficiency of PhoR and ResE kinases with different concentrations of FloA and FloT, respectively. Both PhoR and ResE kinases responded similarly to increasing concentrations of their respective flotillins. A slight improvement in their interaction efficiency was observed with lower concentration of flotillins, which improved with medium concentration of the flotillins. Importantly, the BTH assay that produced higher concentration of the flotillins showed a decrease in the interaction efficiency of both kinases. This is consistent with the typical limitation of scaffold proteins, in that higher concentrations of the scaffold titrate signaling partners into separate complexes, thus inhibiting their interaction [46] (Fig 7B), as it has been experimentally shown in the scaffold protein Ste5 in yeast [47] and the JIP1 scaffold human cells [48]. This suggests that bacterial flotillins act as scaffold proteins to specifically facilitate signal transduction through tethering of signaling partners.

**Fig 8:**
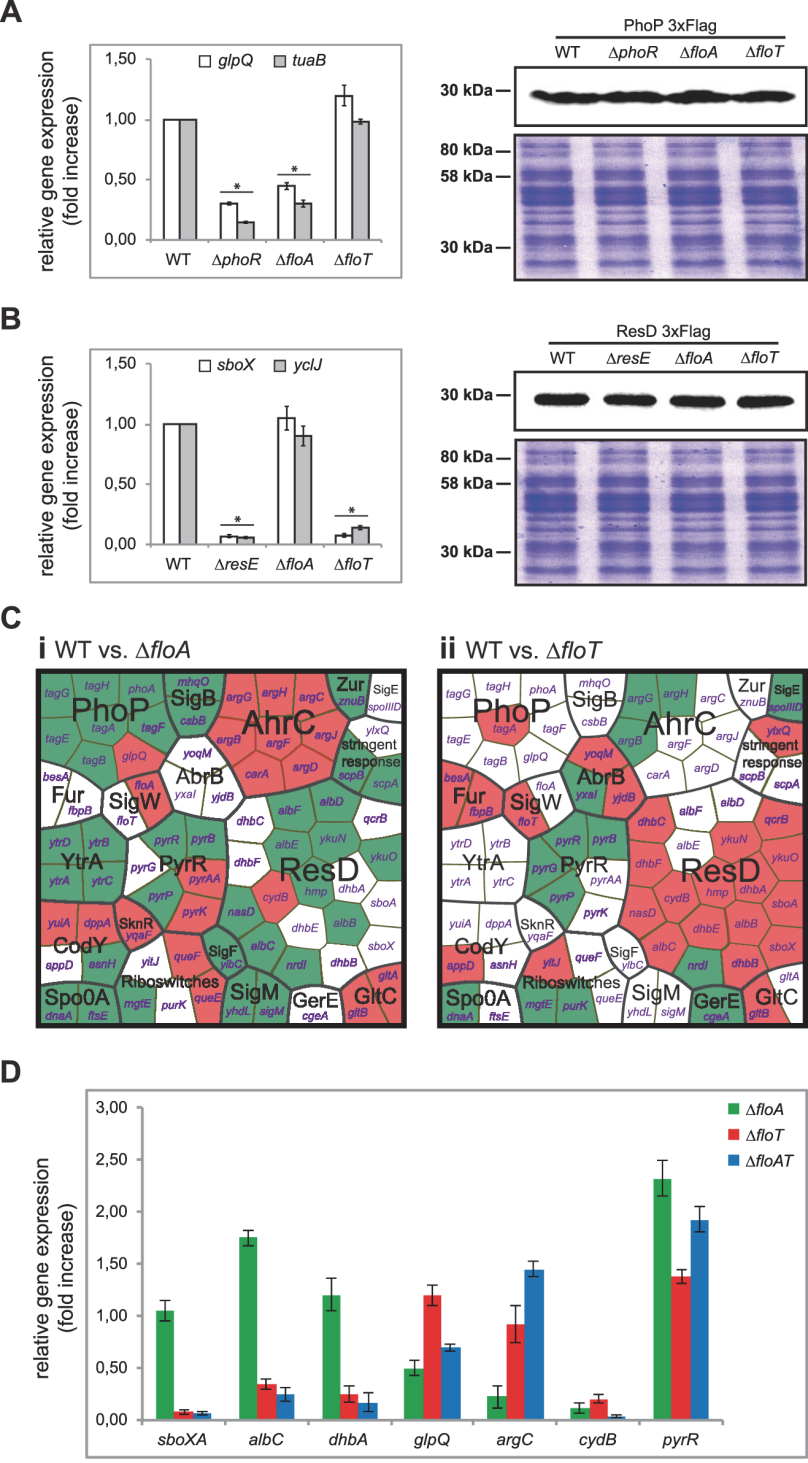
Flotillins influence kinase-dependent activation of PhoP and ResD regulators. **(A)** Left panel shows qRT-PCR of the PhoP-regulated genes *glpQ* and *tuaB* in different genetic backgrounds. Statistically significant differences are marked with an asterisk (Student’s t-test *p* ≤ 0.05). Right panel shows immunoblot assay to detect PhoP-3xFlag in different genetic backgrounds. **(B)** Left panel shows qRT-PCR of the ResD-regulated genes *sboX* and *yclJ* in different genetic backgrounds (Student’s t-test *p* ≤ 0.05). Right panel shows immunoblot assay to detect ResD-3xFlag in different genetic backgrounds (right panel). **(C)** Genome-wide gene expression analysis of flotillin-regulated genes. Voronoi treemaps represent upregulated genes (green sectors) and downregulated genes (red sectors) in the ∆*floA* mutant (i) and ∆*floT* mutant (ii) in comparison to the wild-type strain. Genes whose expression was altered are represented and functionally classified in regulons. Each section is labeled with the name of the genes that represents. **(D)** qRT-PCR analysis of selected genes from the genome-wide gene expression analysis to validate the microarray data.

To investigate the influence of flotillins in the activation of PhoPR and ResDE TCS, we performed qRT-PCR analysis to quantify the transcription of genes which expression is strongly controlled by PhoP and ResD regulators (Fig 8A and 8B). We detected that the expression of the PhoP-regulated genes *glpQ* and *tuaB* involved in cell envelope metabolism [49,50] were reduced in a strain lacking the kinase PhoR and a strain lacking FloA. Likewise, the expression of the ResD-regulated gene *sboX*, responsible for the production of the antibiotic subtilosin [51], and *yclJ*, a gene that encodes for a regulatory protein [52] was reduced in a strain lacking the kinase ResE and a strain lacking FloT (Fig 8A and 8B). Control strains producing tagged versions of the cognate regulators (PhoP-3xFlag and ResD-3xFlag) showed comparable level of the regulators among the different strains, suggesting that the deletion of the respective flotillin specifically affects the activity of each cognate regulator, which in turn inhibits the expression of regulated genes.

Activation of the cognate regulators promotes a conformational change that impacts gene expression. Thus, the protein-protein interaction experiments were coupled to an in-depth analysis of the transcriptional profile of *B*. *subtilis* cells lacking *floA* or *floT* genes. The Δ*floA* and Δ*floT* mutants were grown to stationary phase. Total RNA was purified and used to perform microarray analysis using whole-genome *B*. *subtilis* genechips. Experiments were performed in triplicate and genes were considered differentially expressed when ≥2 fold in expression was detected in all replicates. Our microarray analysis indicated 123 genes to be differentially expressed (S4–S6 Tables and GEO database accession number GSE47918). 77 of these genes belong to different signaling regulons of *B*. *subtilis*, which were organized in a Voronoi treemap (Fig 8C). Each sector of the Voronoi treemap represents a gene and is labeled with the name of the gene that it represents. Each section is labeled in a two-color code to denote upregulated genes (in green) and downregulated genes (in red). There is no biological significance associated with the different shapes that are assigned to each sector. The magnitude of the fold change can be examined in supplemental S4–S6 Tables. This categorization revealed a group of genes whose expression depended on *floA* expression and a second group whose expression depended on *floT* expression. For instance, cells lacking *floA* showed induction of a large number of genes related to cell envelope metabolism, represented by *sigM* and *yhdl*, *yhdK*, *yfml* and *csbB* and *ytrGABCDEF sigM*-induced genes [53]. Additional genes related to cell wall reorganization were also detected (*ytgP*, *dnaA*, *scpA* and *scpB*), including *tagAB* and *tagDEFGH* operons, which are known of being repressed by PhoPR. Cells lacking *floT* displayed a strong inhibition of the genes that constitute the ResDE regulon (*qcrABC*, *ykuNOP*, *dhbABCEF*, *hmp*, *nasDE and sboXA-albABCDEFG*) [54]. Their expression is particularly prominent at stationary phase, when the production of the antibiotic subtilosin (*sboXA-albABCDEFG*) [51] and the siderophore bacillibactin (*dhbABCEF*) [55] is necessary. To validate the results obtained by microarray analysis, we performed qRT-PCR gene expression analysis on several genes that belong to the different regulons that are represented in the Voronoi treemap. qRT-PCR analysis showed comparable results to microarray analysis (Fig 8D).

**Fig 9:**
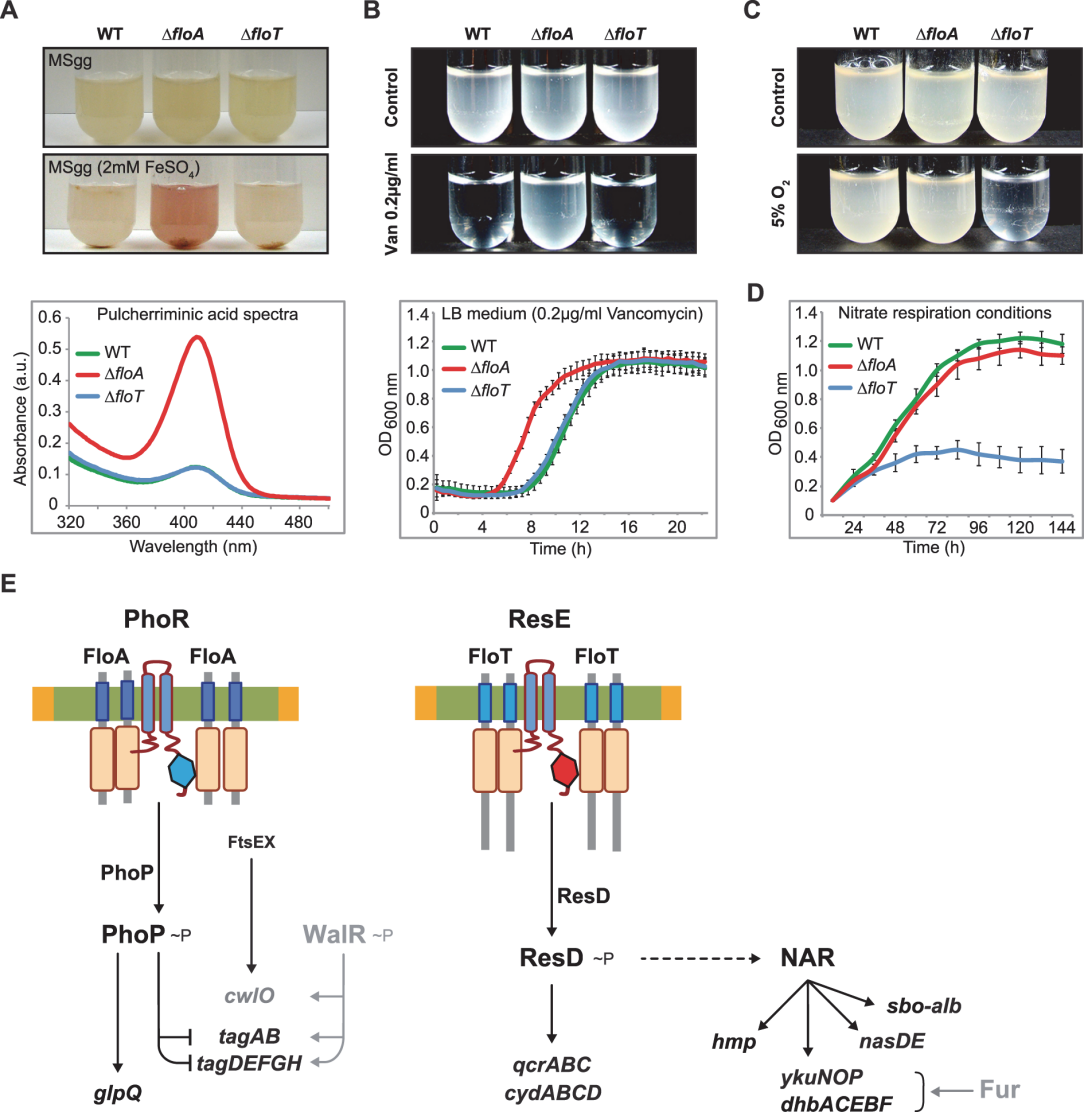
FloA and FloT flotillins influence different physiological processes. **(A)** Upper panel shows different strains grown in liquid MSgg medium and MSgg supplemented with 2mM FeSO_4_ to stationary phase. Bottom panel shows the spectrum of pulcherriminic acid that was measured in cell extracts. **(B)** Upper panel shows the pictures of cultures of different strains that were grown with or without vancomycin (0,2 μg/ml) (MSgg liquid medium). Bottom panel shows their growth curve in the presence of vancomycin (0,2 μg/ml) (LB liquid medium). **(C)** Pictures of cultures of different strains that were grown in MSgg liquid medium in regular atmosphere or under oxygen-limiting conditions. Cells were incubated at 30°C for 72h. **(D)** Growth of the different strains in the absence of oxygen. MSgg medium was modified by replacing glutamate and glycerol with glucose 1% and NaNO_3_ 0,2% **(E)** Schematic representation of the specific signaling pathways that are associated with FloA or FloT. Genes detected in the microarray analysis whose expression was influenced by flotillins are represented in black.

The differential regulation of gene expression that is caused by the activity of FloA and FloT was manifested at the physiological level. We detected phenotypic differences in the Δ*floA* and the Δ*floT* mutants that may be related to the different expression of the controlled genes. For instance, when mutants were grown in Fe^2+^-containing growth medium, only the Δ*floA* mutant accumulated the extracellular red pigment pulcherrimin (Fig 9A), resulting from the condensation of Fe^2+^ with the dipeptide pulcherriminic acid (Leu-Leu) (abs 420 nm) [56]. Pulcherriminic acid accumulates and is released into the medium in response to an excess of amino acid residues that decorate peptidoglycan precursors of bacterial cell wall synthesis, which is usually indicative of a defective cell wall metabolism [56–59]. This suggest that Δ*floA* mutant is defective in cell wall turnover and is consistent to our proteomic and transcriptomic analyses, suggesting that FloA plays a role in the regulation of cell wall metabolism. Moreover, the Δ*floA* mutant showed reduced sensitivity to the antibiotic vancomycin (Fig 9B), similar to other cases in which reduced sensitivity to vancomycin has been observed in cell-wall deficient strains. Vancomycin binds to the C-terminal D-Ala-D-Ala sequence of the pentapeptide peptidoglycan, thereby preventing the integration of peptidoglycan subunits into the cell wall. Cells that show a defective peptidoglycan turnover also show a reduced number of targets to the action of vancomycin and therefore, reduced sensitivity to the action of this antibiotic [60–62]. However, a defective cell wall often implies a less efficient barrier against the diffusion of other antibiotics [63–65], as is the case of the membrane pore-former sublancin [66]. Accordingly, the Δ*floA* mutant shows a higher sensitivity to the glycopeptide sublancin [67].

Likewise, we tested the capacity of the Δ*floA* and Δ*floT* mutants to adapt to stress-related conditions that are typically associated with cultures that undergo stationary phase. When we grew the Δ*floA* and Δ*floT* mutants under oxygen-limiting conditions, only the Δ*floT* mutant displayed a defective growth (Fig 9C). In contrast, the Δ*floA* mutant was able to grow at similar rate to the wild-type strain. The incapacity of the Δ*floT* mutant to adapt to oxygen-limiting conditions could be attributed to a defective activation of the ResDE regulon, as the activation of this regulon is necessary to allow nitrate respiration and thus, cell growth in oxygen-limiting conditions. Our data shows that this mechanism seemed defective only in the Δ*floT* mutant, which grew poorly in oxygen-limiting conditions (Fig 9D). This is consistent with the role that FloT plays in the regulation of stationary phase and stress-related cellular processes, including the activation of the ResDE regulon, which we have detected in our proteomic and transcriptomic data. Taken together, Fig 9E shows a tentative model that integrates our proteomic, transcriptomic and physiological data. This model shows how FloA and FloT scaffold tether distinct signal transduction pathways, which ultimately control different cellular processes in *B*. *subtilis*. Furthermore, this model illustrates how functionally different FMMs regulate different genetic networks in a bacterial cell, which leads to the activation of different physiological processes.

## Discussion

There is growing recognition of the importance of eukaryotic lipid rafts in numerous cellular processes as diverse as protein sorting, membrane trafficking, compartmentalizing signaling cascades or pathogen entry [2,68]. This functional diversity is currently attributed to a different lipid and protein composition of lipid rafts, as it is hypothesized that a heterogeneous population of lipid rafts could exist on cellular membranes specialized on different biological processes [3–5]. Yet, the molecular mechanisms by which cells generate and regulate raft heterogeneity are still unclear. Nevertheless, it is assumed that cells likely regulate the process of raft diversification, to avoid the assembly of membrane signaling platforms that could simultaneously send distinct and conflicting signals to the cell. Here we use a bacterial model to show that *B*. *subtilis* cells are able to diversify FMMs into distinct families of signaling platforms, which are specialized in regulating distinct cellular processes, supporting the current hypothesis that a heterogeneous population of functionally specialized microdomains could exist on cellular membranes.

The discovery of the existence of FMMs adds to other examples of compartmentalization of macromolecules in bacteria, which demonstrate that bacteria are sophisticated organisms with an intricate cellular organization [69,70]. The biological significance of bacterial FMMs could be similar to the role of lipid rafts in eukaryotic cells. One possible function of FMMs could be the generation of a specific microenvironment to protect certain biological processes from inadequate conditions and non-specific interactions. For instance, spatial separation of signal transduction pathways may benefit their interaction specificity. Another plausible role for FMMs is to serve as platforms that control the assembly of membrane-bound protein complexes. By accumulating functionally related proteins in subcellular compartments, the likelihood of interaction increases and thus protein-protein interactions can be efficiently organized in space and time [2,11]. This phenomenon is facilitated by the activity of flotillins, which are FMMs-localized scaffold proteins that coordinate the physical assembly of protein interaction partners [44].

FloA and FloT seem to behave like other scaffold proteins that were described in eukaryotic cells, by specifically tethering signaling partners at lower concentrations or titrating, and thereby inhibiting their interaction at higher concentrations [44,46–48]. We show in this report that FloA and FloT self-interact and distinctively distribute within the FMMs of *B*. *subtilis*. Furthermore, FloA and FloT bind to and facilitate the interaction of different protein components and thus, activate different signal transduction cascades. The main force involved in generating raft heterogeneity is the uneven spatio-temporal distribution of two distinct flotillins FloA and FloT, Similarly, there are two flotillin paralogs in metazoans, FLO-1 and FLO-2, which show differential expression in distinct tissues, suggesting that these proteins may display certain level of specialization in scaffolding distinct cellular processes [71]. Based on this, it is possible that distinct families of lipid rafts may exist in the membrane of eukaryotic cells as well, yet this hypothesis still needs to be experimentally addressed.

Why do cells need or use different rafts? Cells may use this strategy to deliberately activate diverse cellular processes in time to ultimately dictate cell fate [3,72]. Here we show an example in which FMM remodeling occurs during bacterial growth using differential regulatory programs for flotillin expression. While FloA is constitutively expressed, the expression of FloT is restricted to stationary phase. Bacteria could use this mechanism to restrict the assembly and activation of particular protein components to stationary phase. Furthermore, the expression of a different scaffolding protein at stationary phase could help to rapidly adapt the signal transduction networks to face new environmental conditions. Bacteria possibly use this strategy to deliberately activate diverse cellular processes in time to ultimately ensure an effective activation of signaling processes during the lifespan of a bacterium [3,72].

Cells control the expression of each flotillin to restrict their expression to the growth stage in which their functionality is necessary. FloA preferentially tethers protein components associated with cell wall turnover and primary metabolism. Consequently, the Δ*floA* mutant shows a defect in cell wall turnover. In contrast to FloA, FloT is responsible for tethering protein components that are related to adaptation to stationary phase, such as production of siderophores and antibiotics. In addition to this, we found several proteins associated with the FMMs that interact with both FloA and FloT and are related to biofilm formation and sporulation (see Fig 4). An example of this is the membrane-bound protease FtsH that is required for biofilm formation and sporulation [42], which has been shown to interact with FloA and FloT [13,16,17], as we confirmed in this report. Based on these results, it is likely that the Δ*floA* Δ*floT* double mutant shows additional and more pleiotropic defects in signal transduction than the Δ*floA* and Δ*floT* single mutants [17]. Likewise, a pleiotropic defect in cell division and biofilm formation has been associated with the overproduction of both FloA and FloT, which is not observable with the overproduction of either FloA or FloT separately [16].

The differential distribution of flotillin within lipid rafts opens additional questions as to whether other structural components of the lipid rafts, like for instance the constituent lipids, show a different spatio-temporal distribution pattern and thus, may also contribute to raft heterogeneity. All these questions were hindered by the difficulty to characterize subcellular structures in the past. However, the development of recent technologies is changing our knowledge about the structure and function of subcellular structures, including lipid rafts [73,74]. The development of super-resolution microscopes and corresponding data analysis methods may well ease the study of bacteria and offer a tractable model to study the role of membrane microdomains, which is rather complicated in their eukaryotic counterparts. The finding that bacteria organize membrane microdomains functionally and structurally equivalent to lipid rafts represents a remarkable level of sophistication in the organization of bacterial signaling networks that allow prokaryotes to amplify and integrate diverse stimuli. Overall, the spatio-temporal organization of signaling networks in bacteria evidences that bacteria are more complex organisms than previously appreciated.

## Materials and Methods

### Strains, media and growth conditions

*Bacillus subtilis* undomesticated wild type NCIB 3610 was used as parental strain in this study [23]. *Escherichia coli* DH5α and *B*. *subtilis* 168 strains were used for standard cloning and transformation procedures. A full strain list is shown in S1 Table. Selective LB agar was supplemented with antibiotics at final concentrations of: ampicillin 100 μg/ml; spectinomycin 100 μg/ml; erythromycin 2 μg/ml and lincomycin 25 μg/ml, tetracycline 5 μg/ml; chloramphenicol 3 μg/ml; kanamycin 50 μg/ml. When required, surfactin (Sigma, USA) was added from a stock solution to a final concentration of 5 μM. To maintain *B*. *subtilis* cells at exponential phase, cells were grown in shaking liquid LB cultures at 37° C overnight. Liquid LB medium was inoculated with 1:100 volume of the overnight culture and grown to OD600nm = 0.3 with vigorous shaking (200 rpm). To prolong growth at exponential phase, cells were repeatedly passed to fresh LB medium. Passaging was performed when cells reached OD600nm = 0.3. We repeated this procedure as described in [24] for approximately 20 generations prior to cell examination. To search for regulatory proteins that control the expression of *floA* and *floT* genes, the collection of mutants harboring the P_*floA*_-*yfp* and P_*floT*_-*yfp* transcriptional reporters were grown overnight in LB medium at 37°C with continuous agitation (200 rpm). After this, 2 μl of the overnight LB culture was spotted on MSgg agar plates and colonies were allowed to grow at 30°C for 72 h. Images were taken on a Nikon SMZ 1500 Zoom Stereomicroscope equipped with an AxioCam color (Zeiss, Germany). To monitor gene expression, YFP reporter signals were detected using a 520/20 excitation and BP535/30 emission filter. The excitation time was set to 5 s. Unlabeled wild type strain was used as negative control to determine the background.

### Construction of strains

Deletion mutants were generated using long flanking homology PCR [75] (using the primers listed in S3 Table). Markerless gene deletions were used to generate the ∆*floA*, ∆*floT* and ∆*floA* ∆*floT* mutants. Upstream and downstream regions of the *floA* and *floT* genes were joined by long flanking homology PCR [75] and cloned into the vector pMAD [76]. Gene deletion occurs via a sequential process of double recombination. Isolation of the mutants was achieved by counterselection, as described in [76]. The strains harboring the P_*floA*_-GFP and P_*floT*_-GFP transcriptional reporters were generated by cloning the promoter region of *floA* and *floT* into the vector pKM003 containing the *gfp* gene and integrating the constructs into the bacterial genome at the *amyE* locus. The vector pKM003 was kindly provided by Dr. David Rudner (Harvard Medical School, USA).

Translational fusions were constructed by long flanking homology PCR and subsequently cloned into pDR183 or pKM003. The vector pDR183 was kindly provided by Dr. David Rudner. These plasmids allowed the integration of the constructs into the bacterial genome at the *lacA* and *amyE* locus, respectively. Unless specified in the body of the paper, the translational fusions were expressed under the control of their natural promoters. When overexpression of FloA or FloT was necessary (e. g. BN-PAGE), *floA* and *floT* genes were cloned in the pDR111 plasmid under the expression control of an IPTG-inducible promoter P_*hp*_ [77–79]. The constructs were integrated into the bacterial genome at the *amyE* locus. Linearized vectors were added to *B*. *subtilis* 168 cells grown in competence inducing conditions. Double recombination occurred at the *amyE* locus when using the plasmids pDR111 and pKM003 or the *lacA* locus when using the plasmid pDR183. Cells were plated on corresponding selective media and colonies were checked for integration of constructed fusions by colony PCR. Utilizing the same strategy, GFP translational fusions of the kinases ResE and PhoR were generated using the vector pSG1154 and placed under the expression control of a constitutive promoter. SPP1 phage transduction was used to transfer constructs from *B*. *subtilis* 168 to wild type NCIB 3610, according to [80].

Site-directed mutagenesis of the EA C-terminal repeats of FloA and FloT was performed by using an overlap extension PCR. We used an adaption of the protocol that is published in [81]. Complementary primers that harbored the desired mutation were generated and used to amplify *floA* and *floT* genes in combination with outer primers (S2 Table). Two DNA fragments resulted from each gene were subsequently joined to one single fragment using long flanking homology PCR. The resulting gene was further sequenced to confirm the presence of the mutation. Mutations replaced glutamic acid by leucine and alanine by glycine in each specified EA repeat. The resultant variants were fused to GFP or mEOS2 and cloned into pDR183 under the expression control of their own promoter. This allowed the integration of the constructs into the bacterial genome at *lacA* locus by a single event of double recombination.

### Fluorescence microscopy and image analysis

Cells were collected from the cultures by centrifugation, resuspended in 500 μl paraformaldehyde (4%) and incubated 7 min at room temperature to effect fixation. Samples were then subjected to three washing steps and resuspended in PBS buffer. Samples were finally mounted on microscope slides with thin agarose pads (0.8% agarose in PBS). Variations of growth conditions and preparation methods are specified in figure legends. Images were taken on a Leica DMI6000B inverted microscope. The microscope is equipped with a Leica CRT6000 illumination system, a HCX PL APO oil immersion objective with 100 x 1.47 magnification, a Leica DFC630FX color camera and an environment control system. The following filters were used to detect fluorescence signals: BP480/40 excitation filter and BP527/30 emission filter to detect GFP, BP546/40 excitation filter and BP600/40 emission filter to detect mCherry. GFP and mCherry fluorescence was observed by applying excitation times between 100 and 200 ms, while transmitted light images were taken at 36 ms exposure. Leica Application Suite Advanced Fluorescence V3.7 was used to process raw data and fluorescence signals were deconvoluted using AutoQuant software (MediaCybernetics). Further processing of images and calculation of Pearson’s correlation coefficient were performed using ImageJ. To calculate Pearson’s correlation coefficient, we selected 200 cells that simultaneously expressed both kinase and flotillin signals. Pearson’s correlation takes into consideration PhoR/ResE clusters and estimate whether flotillins clusters colocalize with them.

### Photoactivated localization microscopy (PALM)

PALM was performed as described elsewhere [32]. Briefly, we used an inverted microscope (Olympus IX-71) equipped with an oil-immersion objective (60x, NA 1.45; Olympus) [82]. A 405 nm diode laser (Cube 405–100C, Coherent, USA) was used for converting mEOS2 from the green to the red fluorescent state, and a 568 nm laser (Sapphire 568 LP; Coherent, USA) was used for excitation of the converted state. A dichroic mirror (FF580-FDi01-25x36, Semrock, USA) in the excitation path and two emission filters (ET 575 LP, Chroma and FF01-630/92, Semrock, USA) in the detection path were used to image the fluorescence light with an electronmultiplying CCD camera (EMCCD; Ixon DU897, Andor, USA). A pixel size of 106 nm was achieved by using additional lenses. About 20000 frames were recorded with a frame rate of 10 or 20Hz at an excitation intensity of 5kW/cm² (568nm) until no mEOS2 signals could be detected any further. For photoconversion, the 405nm laser was pulsed with a frequency matching the frame rate, with a pulse duration between 1 and 50ms at irradiation intensities of <1 kW/cm². The PALM image stacks were analyzed with the open source software rapidSTORM [83,84], version 2.21. Only single spot events with more than 250 photons were used for image reconstruction. mEOS2 protein fluorescent in consecutive frames was summarized with the "track emissions" filter of rapidSTORM in order to be localized only once and improve localization precision.

Cluster analyses were performed by an in house written python routine (python 2.7.3, Python Software Foundation). The position of one mEOS2 fluorophore was determined as a single localization according to our software rapidSTORM by fitting a Gaussian function to the Point Spread Function. Clusters were defined by either one connected pixel area in image-based analysis or by a cloud of scattered localizations with spatial coherence in localization. Spatial coherence implies that the increased local density of localizations follows a Gaussian distribution within the cluster, which is indicative of the nonrandom distribution of localizations. Using the untracked localization raw data set and the corresponding super-resolved image, a mask was generated to define possible cluster candidates and separate them from the localization pseudo background. A nearest neighbor based global density threshold was applied to assist the separation process, i.e. all localizations exhibiting a nearest neighbor distance above 50 nm were pre-discarded. According to the mask, the tracked localization data set was then filtered by cropping single cluster candidates and rejecting those with just two or less remaining tracked localizations. Cluster diameters where determined by calculating the standard deviation of the localization cloud from its center of mass. The stated cluster diameters represent the FWHM, which was derived from the standard deviation.

### Flow cytometry

*B*. *subtilis* strains harboring translational fusions were grown overnight in LB agar. 3 ml liquid MSgg was inoculated with cells from the overnight culture and grown to stationary phase (OD600nm = 3.5). Next, cells from 1 ml MSgg culture were collected by centrifugation and resuspended in PBS buffer. To disperse cells, sonication was applied in three series of 10 pulses (power output 0.72 / cycle 50%). Finally, cells were diluted 1:200 in PBS buffer and used for analysis. Flow cytometry experiments were conducted using a benchtop MACSQuant Analyzer (Miltenyi Biotech, Germany). Single cells were detected in two scatter channels (FSC, SSC) and one fluorescence channel (B1). Cells were excited by the blue laser (488 nm) coupled to a 488/10 nm filter and detected as size (FSC) and granularity (SSC) signals. GFP fluorescence (B1) was detected by excitation of cells with the blue laser (488 nm) coupled to a 525/50 nm filter. The voltage intensity of channels was set as follows: forward scatter channel (FSC) 265 V, sideward scatter channel (SSC) 410 V and B1 channel 450 V. The number of events measured per sample was 50,000. We used a flow rate of 1,500 to 3,000 events per second. No gates were selected in any experiment. Flow cytometry data was processed with FlowJo 9.4.3 software.

### Whole-genome microarray analysis

To compare the differential transcript levels in the Δ*floA*, Δ*floT* single and Δ*floT* Δ*floA* double mutants, cells were grown in MSgg medium until the late exponential phase and their transcriptome was compared to that of the wild type strain NCIB 3610. Up- and downregulated genes are listed in S3–S5 Tables (Bayes p value ≤ 1.0 x 10–3). The microarray data has been validated for various genes using quantitative RT-PCR experiments (Fig 8). The isolation of total RNA, cDNA synthesis, hybridization, scanning, data normalization has been performed as described previously using three independent biological replicates for each strain [85]. Briefly, pellets were frozen in liquid nitrogen and stored at -80°C. RNA extraction was performed with the Macaloid/Roche protocol [85,86], RNA concentration and purity was measured using NanoDrop ND-1000 Spectrophotometer. RNA samples were reverse transcribed into cDNA using the Superscript III reverse transcriptase kit (Invitrogen, USA) and labeled with Cy3 or Cy5 monoreactive dye (GE Healthcare, The Netherlands). Labeled and purified cDNA samples (Nucleospin Extract II, Biokè, The Netherlands) were hybridized in Ambion Slidehyb #1 buffer (Ambion Europe Ltd) at 48°C for 16 h. The arrays were constructed according to [87]. Briefly, specific oligonucleotides for all 4,107 open reading frames of *B*. *subtilis* 168 were spotted in triplicate onto aldehyde-coated slides (Cell Associates) and further handled using standard protocols for aldehyde slides. Due to the array design, the transcript levels of the plasmid-encoded genes of *B*. *subtilis* 3610 are not determined. Slide spotting, slide treatment after spotting and slide quality control were done as before [88]. After hybridization, slides were washed for 5 min in 2x SSC with 0.5% SDS, 2 times 5 min in 1x SSC with 0.25% SDS, 5 min in 1x SSC 0.1% SDS, dried by centrifugation (2 min, 2.000 rpm) and scanned in GenePix 4200AL (Axon Instruments, USA). Fluorescent signals were quantified using ArrayPro 4.5 (Media Cybernetics, USA) and further processed and normalized with MicroPrep [89]. CyberT [90] was used to perform statistical analysis. Genes with a Bayes P-value of ≤ 1.0 x 10^–4^ were considered significantly affected.

Quantitative PCR experiments were performed as described before [91]. Gene classification was adapted from [92,93]. Data processing in Voronoi treemap was performed with TreeMap software (Macrofocus GMbH, Switzerland). RNA samples obtained as described above for the microarray experiments were treated with RNase-free DNase I (Thermo Fisher Scientific, Germany) for 60 min at 37°C. Reverse transcription was performed with 50 pmol random nonamers on 2 μg of total RNA using RevertAidTM H Minus M-MuLV Reverse Transcriptase (Thermo Fisher Scientific, Germany). Quantification of cDNA was performed on an iQ5 Real-Time PCR System (BioRad, USA) using Maxima SYBR Green qPCR Master Mix (Thermo Fisher Scientific, Germany). We performed 8 replicates reactions per gene analyzed. The primers used are listed in S2 Table. The amount of target cDNA was normalized to the level of *girB* cDNA [94].

### BN-PAGE and immunoblotting

To overexpress the His-tagged version of flotillins (S1 Table), cells from a freshly streaked LB agar plate were used to inoculate 100 ml liquid MSgg medium supplemented with 1 mM IPTG. Cultures were incubated overnight at 37°C with agitation (200 rpm). Cells were collected by centrifugation, resuspended in buffer H [95] containing 1 mM PMSF and lysed in a French pressure cell at 10,000 psi. Cell debris was removed by standard centrifugation at 12,000 x g for 10 min. Membranes were isolated from the supernatant by ultracentrifugation at 100,000 x g, 4°C for 1 h. Pellets containing membranes were carefully resuspended in solubilization buffer A, supplemented with 10% glycerol and 1 mM PMSF protease inhibitor [37]. Samples were subjected to one step of shock freezing in liquid nitrogen and thawed on ice. Membranes were solubilized using 1% dodecyl maltoside (DDM—Glycon Biochemicals, Germany) and prepared for blue native PAGE as described by [37]. To separate protein complexes, samples were mounted on a 4–20% Roti-PAGE gradient gel (Carl Roth, Germany) and blue native PAGE was run for 3 h at 15 mA. Native gels were used for standard immunoblotting procedures without further processing. After blotting, PVDF membranes were destained to eliminate Coomassie staining and washed with TBS-T. His-tagged flotillins were detected using a polyclonal anti-His antibody (MicroMol, Germany). Bands of native complexes that contained the proteins of interest were cut and analyzed by mass spectrometry (LC/MS).

Peptides identified by LC/MS were aligned to the *B*. *subtilis* proteome by using MASCOT Peptide Mass Fingerprint software http://www.matrixscience.com. The protein libraries used for peptide alignment were Uniprot-Swissprot http://www.uniprot.org/, NCBInr http://www.ncbi.nlm.nih.gov/protein, EST-EMBL http://www.ebi.ac.uk/ena/, and Subtilist http://genolist.pasteur.fr/SubtiList/. Alignment conditions were restricted to significance threshold p < 0.05 and ions score cut-off = 15. Protein mass was unrestricted, peptide mass tolerance was 10 ppm and the fragment mass tolerance was 0.02 Da. We systematically discarded proteins that were identified with less than 20% of amino acid coverage or MudPIT score below 700. Even if peptides match randomly, it is possible to obtain multiple matches to a single protein. MudPIT score is a Poisson distribution that defines a threshold to discard random matches based on the ratio between the number of spectra and the number of entries in the database. For MudPIT results, the score for each protein is the amount of peptides (peptide abundance) that are above the threshold.

### Bacterial two-hybrid analysis

*floA* and *floT* genes as well as the versions of these genes with altered EA-repeats and kinase genes *phoR* and *resE* were PCR-amplified (using primers specified in S1 Table) and cloned into the pKNT25 or pUT18 plasmids (EuroMedex, France). Each one of these plasmids contains a gene that encodes for one of the two catalytic domains of the adenylate cyclase from *Bordetella pertussis* (referred to as T25 and T18 catalytic domains). The genes of interest were cloned and C-terminally fused to the T25 and T18 encoding genes. Plasmids were propagated in the *E*. *coli* BTH101 strain (S2 Table). Positive control was the two oligomers of the leucine zipper GCN4, which are fused into the pKT25-zip and pUT18C-zip plasmids respectively. This was provided by EuroMedex. An *E*. *coli* strain harboring pKNT25 and pUT18 plasmids was used as a negative control (S2 Table). Protein-interaction assays were performed following the protocol previously described by Karimova *et al*. [33]. Experiments that required LB plates 100 μg/ml Ampicillin, 50 μg/ml Kanamycin and 40 μg/ml X-Gal were incubated 48h at 30°C. The appearance of blue product indicated protein interaction that can be monitored and quantified. Quantification of Miller units was performed to monitor the efficiency of protein interactions according to [96].

To assay the scaffold activity of FloA and FloT, the kinase genes *phoR* and *resE* were PCR-amplified and cloned into the pKNT25 and pUT18 plasmids. *phoR* and *resE* were C-terminally fused to the T15 and T18 encoding genes and propagated in *E*. *coli* BTH101 strain. Protein-interaction assays were performed following the protocol previously described by Karimova *et al*. [33] to determine the interaction efficiency between PhoR-PhoR and ResE-ResE. These strains were subsequently used to clone pSEVA modulable plasmids [45] that produce different levels of FloA and FloT. We specifically used pSEVA-621, pSEVA-631 and pSEVA-641 plasmids to produce FloA and FloT at different concentrations. These plasmids contain distinct replication origins and propagate in *E*. *coli* at low, medium and high copy number, respectively. This generates low, medium and high concentration of FloA and FloT in the in bacterial two-hybrid *E*. *coli* strains in which the plasmids are propagated. Experiments that required the propagation of pSEVA vectors were performed in LB medium with 100 μg/ml Ampicillin, 50 μg/ml Kanamycin and 10 μg/ml Gentamicin. Quantification of the Miller units was performed to monitor the efficiency of protein interactions, as it is described in [96].

### Physiological assays

Pulcherriminic acid was estimated by using a method adapted from [56,97]. Cell samples containing pulcherrimin were washed twice with methanol and once with distilled water before extraction with 2M NaOH. The amount of pulcherriminic acid that was converted to the sodium salt turned yellow and could be determined spectrophotometrically as a specific peak in absorbance at 410 nm. To assess the sensitivity of wild-type and flotillin mutant strains to vancomycin, MSgg was supplemented with 0.2 μg/ml Vancomycin. Similarly, strains were grown in LB medium supplemented with 0.2 μg/ml Vancomycin in a Tecan Infinite 200 Pro Microplate Reader at 37°C with agitation (200 rpm). Growth was measured at OD = 595nm. The growth curves shown are a mean of three independent experiments. To grow *B*. *subtilis* in anaerobic conditions (i.e. nitrate respiration), MSgg medium was modified by replacing glutamate with sodium nitrate (0,2%) and glycerol with glucose (1%). Liquid cultures were grown in 2.5 L seal chambers containing Oxoid CampyGen (5% O_2_) or anaerobic atsmosphere generation bags (Oxoid, UK).

### Accession numbers

Gene Expression Omnibus (GEO): Accession database number GSE47918

http://www.ncbi.nlm.nih.gov/geo/query/acc.cgi?token=fxkbdeiukswcgxs&acc=GSE47918

## Supporting Information

S1 Fig (Related to main Fig 1) Differential expression of *floA* and *floT* genes. **(A)** Gene expression profile of *floA* (top panel) and *floT* (bottom panel) genes under 249 different growing conditions, according to the published database [21,22]. Overall dataset can be found at *B*. *subtilis* expression data browser http://migale.jouy.inra.fr. and also at SubtiExpress browser at http://subtiwiki.uni-goettingen.de/apps/expression. The gene expression profiles of different cultures growing in LB medium are marked in blue. The gene expression profiles of cells growing in different MSgg growing conditions are marked in red. Expression levels are given in arbitrary units. Details of growth conditions and gene expression levels are presented on the right. **(B)** Flow cytometry analysis monitoring the expression of P_*floA*_-*gfp* and P_*floT*_ *-gfp* transcriptional fusions at single cell level. *B*. *subtilis* cells harboring the transcriptional fusions were grown in LB medium to exponential phase (upper panel) and MSgg medium to stationary phase (bottom panel). X-axis represents fluorescence signal in arbitrary units and Y-axis represents cell count for each strain (50,000 cells were counted). Variations in the gene expression level were exclusively detected in the channel that monitored P_*floT*_ *-gfp* transcriptional fusion. **(C)** Flow cytometry analysis monitoring the expression of the FloA-GFP and FloT-GFP translational fusions at single cell level. Cells were grown in LB medium to exponential phase (upper panel) or MSgg medium to stationary phase (bottom panel). X-axis represents fluorescence signal in arbitrary units and Y-axis represents cell count for each strain (50,000 cells were counted). Variations in the protein expression level were exclusively detected in the channel that monitored FloT-GFP translational fusion. **(D)** Variation of the *floA* and *floT* gene expression in 29 different regulatory mutants. Regulatory genes known to influence diverse signal transduction cascades were systematically deleted in in *B*. *subtilis* strains labeled with the P_*floA*_-*yfp* and P_*floT*_-*yfp* transcriptional fusions. The resulting strains were examined for their ability to regulate the expression of *floA* and *floT* genes. Cells were grown in MSgg agar plates at 30°C for 72h. Fluorescence images were taken on a Nikon SMZ 1500 Zoom Stereomicroscope equipped with an AxioCam color (Carl Zeiss). The most prominent differences in the expression of *floA* and *floT* genes were detected in the Δ*spo0A* and Δ*abrB* genetic backgrounds, which were subsequently followed up in additional experiments at the gene expression level. We also detected differences in the expression of *floT* in the Δ*dlt* mutant but this could be due to an indirect influence to the activation of Spo0A, as it has been published elsewhere [98].
(EPS)

S2 Fig (Related to main Fig 1) The cell-cell communication signal surfactin activates the expression of FloT via Spo0A. **(A)** Fluorescence microscopy pictures of cells labeled with the FloA-GFP in different growing conditions. Scale bars are 2 μm. Growing conditions did not alter the number of FloA foci per cell. **(B)** Quantification of the number of foci per cell (n = 400 cells) in cells labeled with FloA-GFP at different growing conditions. Cells showed similar number of foci at different growing conditions. **(C)** Fluorescence microscopy picture of cells labeled with FloT-GFP in different growing conditions. Scale bars are 2 μm. Cells that were grown in LB medium showed lower number of foci compared to cells that were grown in MSgg medium or LB medium complemented with surfactin 5 μM. **(D)** Quantification of the number of foci per cell (n = 400 cells) in cells labeled with FloT-GFP at different growing conditions. Cells that were grown in LB medium showed lower number of foci compared to the rest of growing conditions. **(E)** Fluorescence microscopy picture of different strains labeled with FloT-GFP. Scale bars are 2 μm. Cells that lack Spo0A showed a very low number of foci that did not change in medium complemented with surfactin 5 μM. **(F)** Quantification of the number of foci per cell (n = 400 cells) in cells deficient in Spo0A and labeled with FloT-GFP. In the absence of Spo0A the number of FloT foci decreases dramatically and it does not change with the addition of surfactin to the medium.
(EPS)

S3 Fig (Related to main Fig 2) FloA and FloT from *B*. *subtilis* are two different flotillin-like proteins. **(A)** Scheme of human FLO-1 and FLO-2 protein structures (left panel) in comparison to FloA and FloT from *B*. *subtilis* (right panel). The N-terminal region of the human FLO-1 attaches to the cellular membrane using a palmitate residue (represented in blue) and/or a myristate residue that is represented in red in the case of FLO-2. The prohibitin homology domain (PHB or SPFH domain) that is typically present in all flotillin proteins is represented in green. In both, human and bacterial flotillins, the C-terminal region contains four EA repeats. The amino acid sequence of this region is magnified. Each EA repeat region is highlighted in orange for better visualization. Scale bar is 100 amino acids. The N-terminal region of the FloA and FloT bacterial flotillins attaches to the cellular membrane using a membrane-anchoring domain that directly binds to the cellular membrane (represented in blue). **(B)** Fluorescence microscopy pictures of fields of cells labeled with the translational fusions FloA-GFP (left upper panel) and FloT-GFP (right upper panel) and FloA-mCherry (left bottom panel) and FloT-mCherry (right bottom panel). Both FloA and FloT flotillin proteins distribute in foci across the bacterial membrane, yet the number of foci organized by FloA is more abundant than the number of foci organized by FloT. Cells were grown in MSgg at 37°C to early stationary phase. Scale bar is 4μm.
(EPS)

S4 Fig (Related to main Fig 3) Subcellular organization of FloA and FloT at high-resolution level. **(A and B)** Fluorescence microscopy pictures at high-resolution level using super-resolution imaging by photoactivated localization microscopy (PALM). In this case, FloA and FloT were fused to the photoactivatable monomeric protein PAmCherry and expressed in *B*. *subtilis* cells. This is a photoactivatable monomeric red fluorescent protein derived from the classical mCherry fluorescent protein. FloA-PAmCherry (Panel A) and FloT-PAmCherry (Panel B) show distribution in foci across the membrane. Clusters are indicated with white arrows. A definition of cluster is in the body of the paper. Data obtained by using PAmCherry is consistent with the data obtained by using fluorescence microscopy and mEOS2/PALM microscopy and validates the distribution of foci presented in main Fig 3. Scale bar is 500 nm. Panel A shows a detail of a dashed-line decorated PALM picture of a FloA-PAmCherry expressing cell as a general indicator of the cell outline. Scale bar is 500 nm. Panel B shows a detail of a dashed-line decorated PALM picture of a FloT-PAmCherry expressing cell as a general indicator of the cell outline. Scale bar is 500 nm. **(C)** Western blot analysis to detect FloA-GFP and FloT-GFP proteins. FloT is more abundant than FloA. Cells expressing GFP-tagged variants were expressed in *B*. *subtilis* using the native promoter. Cells were grown in MSgg at 37°C to early stationary phase. Western blot analysis was performed using purified cellular membranes. Detection was performed using polyclonal antibodies against GFP epitope. Positive control is a non-labelled wild type strain. SDS-PAGE is shown as loading control. **(D)** Relative intensity of the signal detected in the western blot analysis.
(EPS)

S5 Fig (Related to main Figs 4 and 5) Alteration of the subcellular organization of FloA and FloT. **(A) (i)** Fluorescence microscopy pictures of a time-lapse experiment using two different *B*. *subtilis* strains labeled with the translational fusions FloA-GFP (upper row) and FloT-GFP (bottom row). FloA and FloT flotillins show highly dynamic properties [15,16]. Fluorescence foci of FloA-GFP and FloT-GFP rapidly reorganize across the bacterial membrane. A white arrow in the bottom row exemplifies the dynamism of the foci. It shows two foci that are able to quickly reorganize into one single focus. For this experiment, cells were grown in MSgg at 37°C to early stationary phase. Time scale is given in seconds. Scale bars are 2μm. **(ii)** Fluorescence microscopy pictures of a time-lapse experiment using two different *Escherichia coli* strains expressing the translational fusions FloA-GFP (upper row) and FloT-GFP (bottom row). Fluorescence foci of FloA-GFP and FloT-GFP reorganize across the membrane of *E*. *coli* cells in a comparable organization and dynamic pattern than in *B*. *subtilis* cells. **(B)** Alteration of the EA repeats from the Ct region of FloA and FloT was performed by site-directed mutagenesis. We replaced glutamic acid (E) and alanine (A) amino acids with leucine (L) and glycine (G), respectively. L and G amino acids were chosen for amino acid replacement due to the subtle changes in the structure or polarity of the amino acid backbones, which guarantees a minimum level of interference with protein folding. Differences in amino acid replacement are framed in red for better visualization. **(C)** Fluorescence microscopy pictures evidencing the distribution pattern of FloT (upper row) and FloA (bottom row) in the absence of the alternative flotillin. The absence of one flotillin does not affect the distribution pattern of the other flotillin and the differences that were detected in the number of foci were not significant, according to our statistical analysis (Student’s t-test *p* ≤ 0.05). However, alterations of the EA repeats caused a severe alteration in the distribution pattern of the flotillins. Fluorescence microscopy of cells expressing GFP-tagged versions of FloT and its different EA variants in a *floA*-deficient genetic background (upper row). Fluorescence microscopy of cells expressing GFP-tagged versions of FloA and its different EA variants in a *floT*-deficient background (bottom row). Scale bars are 2μm. Cells were grown in MSgg at 37°C to early stationary phase. **(D)** Western blot analysis of wild-type FloA and FloT expression levels in relation to FloA EA4 and FloT EA2 variants. GFP-tagged version of FloA and FloT variants were expressed in *B*. *subtilis* using their native promoter. Cells were grown in MSgg at 37°C to early stationary phase. Western blot analysis was performed using purified cellular membranes. Flotillin detection was performed using polyclonal antibodies against GFP epitope. Positive control is a non-labelled wild type strain. SDS-PAGE are shown as loading control.
(EPS)

S6 Fig (Related to main Fig 8) The translational fusions PhoR-GFP and ResE-GFP are functional in *B*. *subtilis* cells. **(A)** qRT-PCR of the PhoP-regulated genes *tuaB* in different genetic backgrounds. Δ*phoR* mutant complemented with PhoR-GFP translational fusion recovered the expression of *tuaB* gene. Statistically significant differences are marked with an asterisk (Student’s t-test *p* ≤ 0.05). **(B)** qRT-PCR of the ResD-regulated gene *yclJ* in different genetic backgrounds. Δ*resD* mutant complemented with ResD-GFP translational fusion recovered the expression of *yclJ* gene. Statistically significant differences are marked with an asterisk (Student’s t-test *p* ≤ 0.05).
(EPS)

S1 Table (Related to material and methods) List of strains and plasmids used in this study. (DOCX)

S2 Table (Related to material and methods) List of primers used in this study. (DOCX)

S3 Table (Related to main Fig 6) List of proteins identified in membrane-associated protein complexes that interacted exclusively with either FloA (green squares) or FloT (yellow squares), or FloA and FloT (blue squares). (DOCX)

S4 Table (Related to main Figs 7 and 8) List of genes that are significantly up or downregulated (Bayes.p value <10–4) in the Δ*floA* cells compared to wild-type cells. Mean indicates log 2 transformed expression ratios.
(DOCX)

S5 Table (Related to main Figs 7 and 8) List of genes that are significantly up or downregulated (Bayes.p value <10–4) in the Δ*floT* cells compared to wild-type cells. Mean indicates log 2 transformed expression ratios.
(DOCX)

S6 Table (Related to main Figs 7 and 8) List of genes that are significantly up or downregulated (Bayes.p value <10–4) in the Δ*floA* Δ*floT* cells compared to wild-type cells. Mean indicates log 2 transformed expression ratios.
(DOCX)
