# Supplementary material for: Spatio-temporal Remodeling of Functional Membrane Microdomains Organizes the Signaling Networks of a Bacterium
Source: PLoS Genet. 2015 Apr 24;11(4):e1005140. doi: 10.1371/journal.pgen.1005140 (PMC4409396; doi:10.1371/journal.pgen.1005140)
Supplement: S1 Table — (DOCX) [file pgen.1005140.s007.docx]

**Supplemental Information**

**Supplemental Tables**

**Supplemental Table S1 (Related to material and methods):** List of strains and plasmids used in this study

| Strain | Genotype | Reference |
| --- | --- | --- |
| DL1 | Wild type (NCIB 3610) | [1] |
| DL2 | Wild type 168 | [2] |
| GK129 | 3610 *lacA*::P*_floT_*-*yfp* (*mls*) | This study |
| GK38 | 168 *amyE*::P*_floT_*-*yfp* (*spc*) | This study |
| GK43 | 3610 Δ*srf*::*mls* *amyE*::P*_floT_*-*yfp* (*spc*) | This study |
| GK45 | 3610 Δ*kinC*::*cm* *amyE*::P*_floT_*-*yfp* (*spc*) | This study |
| GK47 | 3610 Δ*kinD*::*tet* *amyE*::P*_floT_*-*yfp* (*spc*) | This study |
| GK49 | 3610 Δ*sigE*::*mls amyE*::P*_floT_*-*yfp* (*spc*) | This study |
| GK51 | 3610 Δ*sigF*::*km* *amyE*::P*_floT_*-*yfp* (*spc*) | This study |
| GK53 | 3610 Δ*spo0A*::*mls* *amyE*::P*_floT_*-*yfp* (*spc*) | This study |
| GK55 | 3610 Δ*comA*::*cm* *amyE*::P*_floT_*-*yfp* (*spc)* | This study |
| GK57 | 3610 Δ*spo0E*::*km* *amyE*::P*_floT_*-*yfp* (*spc*) | This study |
| GK59 | 3610 Δ*dlt*::*tet* *amyE*::P*_floT_*-*yfp* (*spc*) | This study |
| GK61 | 3610 Δ*rapD*::*km* *amyE*::P*_floT_*-*yfp* (*spc*) | This study |
| GK63 | 3610 Δ*rapG*::*cm* *amyE*::P*_floT_*-*yfp* (*spc*) | This study |
| GK65 | 3610 Δ*abrB*::*tet* *amyE*::P*_floT_*-*yfp* (*spc*) | This study |
| GK67 | 3610 Δ*abh*::*km* *amyE*::P*_floT_*-*yfp* (*spc*) | This study |
| GK69 | 3610 Δ*degS*::*tet* *amyE*::P*_floT_*-*yfp* (*spc*) | This study |
| GK71 | 3610 Δ*lgtR*::*cm* *amyE*::P*_floT_*-*yfp* (*spc*) | This study |
| GK73 | 3610 Δ*slr*::*tet amyE*::P*_floT_*-*yfp* (*spc*) | This study |
| GK75 | 3610 Δ*rapH*::*km* *amyE*::P*_floT_*-*yfp* (*spc*) | This study |
| GK77 | 3610 Δ*ftsH*::*km amyE*::P*_floT_*-*yfp* (*spc*) | This study |
| GK109 | 3610 Δ*codY*::*spc* *lacA*::P*_floT_*-*yfp* (*mls*) | This study |
| GK110 | 3610 Δ*mecA*::*spc lacA*::P*_floT_*-*yfp* (*mls*) | This study |
| GK111 | 3610 Δ*compqx*::*spc* *lacA*::P*_floT_*-*yfp* (*mls*) | This study |
| GK112 | 3610 Δ*sinR*::*spc lacA*::P*_floT_*-*yfp* (*mls*) | This study |
| GK113 | 3610 Δ*comK*::*spc* *lacA:*:P*_floT_*-*yfp* (*mls*) | This study |
| GK119 | 3610 Δ*relA*::*km* *amyE*::P*_floT_*-*yfp* (*spc*) | This study |
| GK126 | 3610 Δ*hpr*::*cm* l*acA*::P*_floT_*-*yfp* (*mls*) | This study |
| GK127 | 3610 Δ*spo0F*::*km* *lacA*::P*_floT_*-*yfp* (*mls*) | This study |
| GK128 | 3610 Δ*rapD*::*cm* *lacA*::P*_floT_*-*yfp* (*mls*) | This study |
| GK131 | 3610 Δ*sqhC*::*km* *lacA*::P*_floT_*-*yfp* (*mls*) | This study |
| GK132 | 3610 Δ*rapB*::*spc* l*acA*::P*_floT_*-*yfp* (*mls*) | This study |
| GK133 | 3610 Δ*sinI*::*spc* *lacA*::P*_floT_*-*yfp* (*mls*) | This study |
| GK82 | 168 *amyE*::P_f_*_loA_*-*yfp* (*spc*) | This study |
| GK83 | 3610 *amyE*::P*_floA_*-*yfp* (*spc*) | This study |
| GK116 | 168 *lacA*::P*_floA_*-*yfp* (*mls*) | This study |
| GK84 | 3610 Δ*srf*::*mls* *amyE*::P*_floA_*-*yfp* (*spc*) | This study |
| GK85 | 3610 Δ*kinC*::*cm* *amyE*::P*_floA_*-*yfp* (*spc*) | This study |
| GK86 | 3610 Δ*kinD*::*tet amyE*::P*_floA_*-*yfp* (*spc*) | This study |
| GK87 | 3610 Δ*sigE*::*mls amyE*::P*_floA_*-*yfp* (*spc*) | This study |
| GK88 | 3610 Δ*sigF*::*km amyE*::P*_floA_*-*yfp* (*spc*) | This study |
| GK96 | 3610 Δ*spo0A*::*mls amyE*::P*_floA_*-*yfp* (*spc*) | This study |
| GK99 | 3610 Δ*comA*::*cm amyE*::P*_floA_*-*yfp* (*spc*) | This study |
| GK100 | 3610 Δ*spo0E*::*km amyE*::P*_floA_*-*yfp* (*spc*) | This study |
| GK92 | 3610 Δ*dlt*::*tet amyE*::P*_floA_*-*yfp* (*spc*) | This study |
| GK93 | 3610 Δ*rapD*::*km amyE*::P*_floA_*-*yfp* (*spc*) | This study |
| GK95 | 3610 Δ*rapG*::*cm amyE*::P*_floA_*-*yfp* (*spc*) | This study |
| GK89 | 3610 Δ*abrB*::*tet amyE*::P*_floA_*-*yfp* (*spc*) | This study |
| GK90 | 3610 Δ*abh*::*km amyE*::P*_floA_*-*yfp* (*spc*) | This study |
| GK91 | 3610 Δ*degS*::*tet amyE*::P*_floA_*-*yfp* (*spc*) | This study |
| GK97 | 3610 Δ*lgtR*::*cm* *amyE*::P*_floA_*-*yfp* (*spc*) | This study |
| GK98 | 3610 Δ*slr*::*tet amyE*::P*_floA_*-*yfp* (*spc*) | This study |
| GK94 | 3610 Δ*rapH*::*km amyE*::P*_floA_*-*yfp* (*spc*) | This study |
| GK124 | 3610 Δ*ftsH*::*km lacA*::P*_floA_*-*yfp* (*mls*) | This study |
| GK125 | 3610 Δ*codY*::*spc lacA*::P*_floA_*-*yfp* (*mls*) | This study |
| GK123 | 3610 Δ*mecA*::*spc lacA*::P*_floA_*-*yfp* (*mls*) | This study |
| GK122 | 3610 Δc*ompqx*::*spc lacA*::P*_floA_*-*yfp* (*mls*) | This study |
| GK121 | 3610 Δ*sinR*::*spc lacA*::P*_floA_*-*yfp* (*mls*) | This study |
| GK120 | 3610 Δ*comK*::*spc lacA*::P*_floA_*-*yfp* (*mls*) | This study |
| GK102 | 3610 Δ*relA*::*km amyE*::P*_floA_*-*yfp* (*spc*) | This study |
| GK134 | 3610 Δ*hpr*::*cm lacA*::P*_floA_*-*yfp* (*mls*) | This study |
| GK135 | 3610 Δ*spo0F*::*km lacA*::P*_floA_*-*yfp* (*mls*) | This study |
| GK139 | 3610 Δ*rapD*::*cm lacA*::P*_floA_*-*yfp* (*mls*) | This study |
| GK137 | 3610 Δs*qhC*::*km lacA*::P*_floA_*-*yfp* (*mls*) | This study |
| GK138 | 3610 Δ*rapB*::*spc lacA*::P*_floA_*-*yfp* (*mls*) | This study |
| GK136 | 3610 Δ*sinI*::*spc lacA*::P*_floA_*-*yfp* (*mls*) | This study |
| DL573 | 3610 Δ*spo0A::mls* | [1] |
| DL383 | 3610 Δ*abrB::tet* | [3] |
| JS136 | 3610 *amyE::floA-gfp* (*spc*) | This study |
| JS280 | 3610 *amyE::floT-gfp* (*spc*) | This study |
| JS170 | 3610 Δ*spo0A::mls* *amyE::floA-gfp* (*spc*) | This study |
| JS169 | 3610 Δ*spo0A::mls* *amyE::floT-gfp* (*spc*) | This study |
| JS177 | 3610 Δ*spo0A::mls* Δ*abrB::tet* *amyE::floA-gfp* (*spc*) | This study |
| JS181 | 3610 Δ*spo0A::mls* Δ*abrB::tet* *amyE::floT-gfp* (*spc*) | This study |
| JS183 | 168 *lacA::floT-rfp* (*mls*) | This study |
| JS320 | 168 *lacA::floA-rfp* (*mls*) | This study |
| JS186 | 3610 *lacA::floT-rfp* (*mls*) *amyE::floA-gfp* (*spc*) | This study |
| JS321 | 3610 *lacA::floA-rfp* (*mls*) *amyE::floT-gfp* (*spc*) | This study |
| JS134 | 3610 *lacA::floA-mEos2* (*mls*) | This study |
| JS153 | 3610 *lacA::floT-mEos2* (*mls*) | This study |
| JS119 | 3610 Δ*floT* (markerless) | [4] |
| JS152 | 3610 Δ*floA* (markerless) | [4] |
| JS201 | 3610 Δ*floT* (markerless) *amyE::P_hp_-floT-His^6^* (*spc*) | [5] |
| JS202 | 3610 Δ*floA* (markerless) *amyE::P_hp_-floA-His^6^* (*spc*) | [5] |
| JS303 | 168 *amyE::floT-gfp* [A342G,E343L,A344G] (*spc*) | This study |
| JS304 | 168 *amyE::floT-gfp* [A357G,E358L,A359G,E360L] (*spc*) | This study |
| JS305 | 168 *amyE::floT-gfp* [A370G,E371L,A372G,E373L] (*spc*) | This study |
| JS306 | 168 *amyE::floT-gfp* [A390G,E391L,A392G,E393L,A394G] (*spc*) | This study |
| JS310 | 168 *amyE::floA-gfp* [A240G,E241L,A242G] (*spc*) | This study |
| JS311 | 168 *amyE::floA-gfp* [A251G,E252L,E252L] (*spc*) | This study |
| JS317 | 168 *amyE::floA-gfp* [E278L,A279G,E280L,A281G,E282L] (*spc*) | This study |
| JS312 | 168 *amyE::floA-gfp* [A288G,E289L,A290G] (*spc*) | This study |
| JS334 | 3610 Δ*floT* (markerless) *lacA::floT-mEos2* [A357G,E358L,A359G,E360L] (mls) | This study |
| JS335 | 3610 Δ*floA* (markerless) *lacA::floA-mEos2* [A288G,E289L,A290G] (mls) | This study |
| JS461 | 168 *lacA::floA_T_-gfp* (*mls*) | This study |
| JS470 | 168 *lacA::floT_A_-gfp* (*mls*) | This study |
| JS166 | 3610 *lacA::floT-PAmCherry* (*mls*) | This study |
| JS167 | 3610 *lacA::floA-PAmCherry* (*mls*) | This study |
| BM155 | 168 Δ*floT* (markerless) | This study |
| DL1401 | 168 Δ*floA::mls* | [6] |
| JS338 | 168 Δ*floA::mls* *amyE::floT-gfp* [A342G,E343L,A344G] (*spc*) | This study |
| JS341 | 168 Δ*floA::mls* *amyE::floT-gfp* [A357G,E358L,A359G,E360L] (*spc*) | This study |
| JS339 | 168 Δ*floA::mls* *amyE::floT-gfp* [A370G,E371L,A372G,E373L] (*spc*) | This study |
| JS342 | 168 Δ*floA::mls* *amyE::floT-gfp* [A390G,E391L,A392G,E393L,A394G] (*spc*) | This study |
| JS343 | 168 Δ*floA::mls* *amyE::floT-gfp* (*spc*) | This study |
| JS345 | 168 Δ*floT* (markerless) *amyE::floA-gfp* [A240G,E241L,A242G] (*spc*) | This study |
| JS346 | 168 Δ*floT* (markerless) *amyE::floA-gfp* [A251G,E252L,E252L] (*spc*) | This study |
| JS347 | 168 Δ*floT* (markerless) *amyE::floA-gfp* [E278L,A279G,E280L,A281G,E282L] (*spc*) | This study |
| JS348 | 168 Δ*floT* (markerless) *amyE::floA-gfp* [A288G,E289L,A290G] (*spc*) | This study |
| JS357 | 168 Δ*floT* (markerless) *amyE::floA-gfp* (*spc*) | This study |
| DL1662 | 168 *amyE::P_hp_-phoP-3xFlag* (*spc*) | This study |
| DL1664 | 168 *amyE::P_hp_-resD-3xFlag* (*spc*) | This study |
| DL1666 | 168 Δ*floA::mls amyE::P_hp_-phoP-3xFlag* (*spc*) | This study |
| DL1668 | 168 Δ*floA::mls amyE::P_hp_-resD-3xFlag* (*spc*) | This study |
| DL1670 | 168 Δ*floT* (markerless) *amyE::P_hp_-phoP-3xFlag* (*spc*) | This study |
| DL1672 | 168 Δ*floT* (markerless) *amyE::P_hp_-resD-3xFlag* (*spc*) | This study |
| DL1681 | 168 Δ*phoR::km amyE::P_hp_-phoP-3xFlag* (*spc*) | This study |
| DL1679 | 168 Δ*resE::km amyE::P_hp_-resD-3xFlag* (*spc*) | This study |
| JS506 | 168 *lacA::floT-rfp* (*mls*) *amyE::resE-gfp* (*spc*) | This study |
| JS508 | 168 *lacA::floA-rfp* (*mls*) *amyE::phoR-gfp* (*spc*) | This study |
| JS517 | 168 Δ*resE::km amyE::resE-gfp* (*spc*) | This study |
| JS518 | 168 Δ*phoR::km amyE::phoR-gfp* (*spc*) | This study |
| DL95 | *E. coli* DH5α | [7] |
| JS263 | *E. coli* DH5α pDR183 *P_floT_-floT-gfp* | This study |
| JS314 | *E. coli* DH5α pDR111 *P_floA_-floA-gfp* | This study |
| BM263 | *E. coli* BTH101 | [8] |
| BM261 | *E. coli* DH5α pKT25-*zip* | This study |
| BM262 | *E. coli* DH5α pUT18C-*zip* | This study |
| BM258 | *E. coli* DH5α pKNT25 | This study |
| BM259 | *E. coli* DH5α pUT18 | This study |
| JS369 | *E. coli* BTH101 pKT25-*zip* pUT18C-*zip* | This study |
| JS368 | *E. coli* BTH101 pKNT25 pUT18 | This study |
| JS360 | *E. coli* BTH101 pKNT25-*floT* pUT18-*resE* | This study |
| JS378 | *E. coli* BTH101 pKNT25-*floT* pUT18-*phoR* | This study |
| JS379 | *E. coli* BTH101 pKNT25-*floA* pUT18-*resE* | This study |
| JS362 | *E. coli* BTH101 pKNT25-*floA* pUT18-*phoR* | This study |
| JS370 | *E. coli* BTH101 pKNT25-*floT* pUT18-*floT* | This study |
| JS371 | *E. coli* BTH101 pKNT25-*floT* pUT18-*floA* | This study |
| JS372 | *E. coli* BTH101 pKNT25-*floA* pUT18-*floT* | This study |
| JS373 | *E. coli* BTH101 pKNT25-*floA* pUT18-*floA* | This study |
| JS394 | *E. coli* BTH101 pKNT25-*floT* pUT18-*floT* [A342G,E343L,A344G] | This study |
| JS395 | *E. coli* BTH101 pKNT25-*floT* pUT18-*floT* [A357G,E358L,A359G,E360L] | This study |
| JS380 | *E. coli* BTH101 pKNT25-*floT* pUT18-*floT* [A370G,E371L,A372G,E373L] | This study |
| JS381 | *E. coli* BTH101 pKNT25-*floT* pUT18-*floT* [A390G,E391L,A392G,E393L,A394G] | This study |
| JS396 | *E. coli* BTH101 pKNT25-*floA* pUT18-*floA* [A240G,E241L,A242G] | This study |
| JS397 | *E. coli* BTH101 pKNT25-*floA* pUT18-*floA* [A251G,E252L,E252L] | This study |
| JS382 | *E. coli* BTH101 pKNT25-*floA* pUT18-*floA* [E278L,A279G,E280L,A281G,E282L] | This study |
| JS383 | *E. coli* BTH101 pKNT25-*floA* pUT18-*floA* [A288G,E289L,A290G] | This study |
| JS442 | *E. coli* DH5α pSEVA641 | [9] |
| JS445 | *E. coli* DH5α pSEVA631 | [9] |
| JS446 | *E. coli* DH5α pSEVA621 | [9] |
| JS441 | *E. coli* BTH101 pKNT25-*phoR* pUT18-*phoR* | This study |
| JS443 | *E. coli* BTH101 pKNT25-*resE* pUT18-*resE* | This study |
| JS444 | *E. coli* DH5α pSEVA641 *P_floA_-floA-His^6^* | This study |
| JS447 | *E. coli* DH5α pSEVA631 *P_floA_-floA-His^6^* | This study |
| JS448 | *E. coli* DH5α pSEVA621 *P_floA_-floA-His^6^* | This study |
| JS450 | *E. coli* DH5α pSEVA641 *P_floT_-floT-His^6^* | This study |
| JS451 | *E. coli* DH5α pSEVA631 *P_floT_-floT-His^6^* | This study |
| JS452 | *E. coli* DH5α pSEVA621 *P_floT_-floT-His^6^* | This study |

1. Branda SS, Gonzalez-Pastor JE, Ben-Yehuda S, Losick R, Kolter R (2001) Fruiting body formation by *Bacillus subtilis*. Proc Natl Acad Sci U S A 98: 11621-11626.

2. Moszer I, Glaser P, Danchin A (1995) SubtiList: a relational database for the *Bacillus subtilis* genome. Microbiology 141 ( Pt 2): 261-268.

3. Hamon MA, Stanley NR, Britton RA, Grossman AD, Lazazzera BA (2004) Identification of AbrB-regulated genes involved in biofilm formation by *Bacillus subtilis*. Mol Microbiol 52: 847-860.

4. Yepes A, Schneider J, Mielich B, Koch G, Garcia-Betancur JC, et al. (2012) The biofilm formation defect of a *Bacillus subtilis* flotillin-defective mutant involves the protease FtsH. Mol Microbiol 86: 457-471.

5. Mielich-Suss B, Schneider J, Lopez D (2013) Overproduction of flotillin influences cell differentiation and shape in *Bacillus subtilis*. MBio 4: e00719-00713.

6. Lopez D, Kolter R (2010) Functional microdomains in bacterial membranes. Genes Dev 24: 1893-1902.

7. Reusch RN, Hiske TW, Sadoff HL (1986) Poly-beta-hydroxybutyrate membrane structure and its relationship to genetic transformability in *Escherichia coli*. J Bacteriol 168: 553-562.

8. Karimova G, Pidoux J, Ullmann A, Ladant D (1998) A bacterial two-hybrid system based on a reconstituted signal transduction pathway. Proc Natl Acad Sci U S A 95: 5752-5756.

9. Silva-Rocha R, Martinez-Garcia E, Calles B, Chavarria M, Arce-Rodriguez A, et al. (2013) The Standard European Vector Architecture (SEVA): a coherent platform for the analysis and deployment of complex prokaryotic phenotypes. Nucleic Acids Res 41: D666-675.
