# Supplementary material for: Spatio-temporal Remodeling of Functional Membrane Microdomains Organizes the Signaling Networks of a Bacterium
Source: PLoS Genet. 2015 Apr 24;11(4):e1005140. doi: 10.1371/journal.pgen.1005140 (PMC4409396; doi:10.1371/journal.pgen.1005140)
Supplement: S2 Table — (DOCX) [file pgen.1005140.s008.docx]

**Supplemental Table S2 (Related to material and methods):** List of primers used in this study.

| **Name** | **Sequence (5'-3')** | **Description** |
| --- | --- | --- |
| JS23 | ATGAGTGCGATTAAGCCAGAC | *mEos2* fw |
| JS24 | TTTTGGATCCTTATCGTCTGGCATTGTCAGG | *mEos2* rv (BamHI) |
| JS37 | TTTTCTCGAGTTATCGTCTGGCATTGTCAGG | *mEos2* rv (XhoI) |
| JS13 | TAATGAATTCGTGAGCAGTCAACTGTC | P*_floA_* fw (EcoRI) |
| JS40 | CGGATCCATATAACTTCTCCTCCTTAGCAGATTTGACCAATCCC | P*_floA_* rv (PCR *floA*) |
| JS41 | AAAAGTCGACCGCAGCAGTCAGCTGC | P*_floT_* fw (SalI) |
| JS38 | CATTGTCATATCAAATTCCTCCTTAATCAATGCATTGATGAACGG | P*_floT_* rv (PCR *floT*) |
| JS4 | TTTTGTCGACTAAGGAGGAGATATGACAATGCCGATTAT | *floT* fw (SalI) |
| JS1 | TTTTGCATGCTTACTCTGATTTTTGGATCG | *floT* rv (SphI) |
| JS26 | GTCTGGCTTAATCGCACTCATCTCTGATTTTTGGATCGTTTTGG | *floT* rv (PCR *mEos2*) |
| JS2 | TTTTGTCGACTAAGGAGGATATATGGATCCGTCAACAC | *floA* fw (SalI) |
| JS3 | TTTTGCATGCTTATGATTTGCGGTCTTCA | *floA* rv (SphI) |
| JS29 | GTCTGGCTTAATCGCACTCATTGATTTGCGGTCTTCATCCGAAG | *floA* rv (PCR *mEos2*) |
| JS72 | CCAAAACGATCCAAAAATCAGAGATGAGTAAAGGAGAAGAACTTTTC | *gfp* fw (PCR *floT*) |
| JS73 | GAAAAGTTCTTCTCCTTTACTCATCTCTGATTTTTGGATCGTTTTGG | *floT* rv (PCR *gfp*) |
| BM9 | AAAAGCATGCTTATTTGTATAGTTCATCCATGC | *gfp* rv (SphI) |
| JS52 | CCTCGCCCTTGCTCACCATCTCTGATTTTTGGATCGTTTTGG | *floT* rv (PCR *rfp*) |
| JS101 | CATCTTCTGATGATGCCATTGATTTGCGGTCTTCATCCGAAG | *floA* rv (PCR *rfp*) |
| BM84 | AAAAGCATGCTTACTTGTACAGCTCGTCCAT | *rfp* rv (SphI) |
| JS46 | ATGGTGAGCAAGGGCGAGG | *PAmCherry* fw |
| JS47 | TTTTCTCGAGTTACTTGTACAGCTCGTCCATG | *PAmCherry* rv (XhoI) |
| JS49 | CCTCGCCCTTGCTCACCATAGCAGCTGATTTGCGGTCTTCATCCGAAG | *floA* rv (PCR *PAmCherry*) |
| JS51 | CCTCGCCCTTGCTCACCATAGCAGCCTCTGATTTTTGGATCGTTTTGG | *floT* rv (PCR *PAmCherry*) |
| BM30 | CAAACAGCTCGGGCTAGGCGATGCCAAGAA | *floT* rv EA-Repeat 1 |
| BM31 | TTCTTGGCATCGCCTAGCCCGAGCTGTTTG | *floT* fw EA-Repeat 1 |
| BM32 | AATGGCAAAGGGTCTGGGGCTAAAAGTAAGAA | *floT* rv EA-Repeat 2 |
| BM33 | TTCTTACTTTTAGCCCCAGACCCTTTGCCATT | *floT* fw EA-Repeat 2 |
| BM34 | GCTAGCAAAAGGACTAGGGCTAAAAGCGAAAG | *floT* rv EA-Repeat 3 |
| BM35 | CTTTCGCTTTTAGCCCTAGTCCTTTTGCTAGC | *floT* fw EA-Repeat 3 |
| BM36 | CCTGAAAGGTCTTGGACTAGGGCTAGGAAAAGAGAAAATT | *floT* rv EA-Repeat 4 |
| BM37 | AATTTTCTCTTTTCCTAGCCCTAGTCCAAGACCTTTCAGG | *floT* fw EA-Repeat 4 |
| BM41 | AACCGATCAGGGCCTGGGTGATAAAAACAT | *floA* rv EA-Repeat 1 |
| BM42 | ATGTTTTTATCACCCAGGCCCTGATCGGTT | *floA* fw EA-Repeat 1 |
| BM43 | GCAGGCAAAAGGGCTACTACGACGTGCGAT | *floA* rv EA-Repeat 2 |
| BM44 | ATCGCACGTCGTAGTAGCCCTTTTGCCTGC | *floA* fw EA-Repeat 2 |
| BM45 | GAAAGTAGTACTAGGCCTGGGGCTAGTGCCGCTTG | *floA* rv EA-Repeat 3 |
| BM46 | CAAGCGGCACTAGCCCCAGGCCTAGTACTACTTTC | *floA* fw EA-Repeat 3 |
| BM47 | GCTTGCGATGGGACTAGGTTTGCGTGAAGG | *floA* rv EA-Repeat 4 |
| BM48 | CCTTCACGCAAACCTAGTCCCATCGCAAGC | *floA* fw EA-Repeat 4 |
| JS103 | TTTTGCATGCATGACAATGCCGATTATAATGATC | *floT* fw (ShI) |
| JS104 | TTTTGGTACCCGCTCTGATTTTTGGATCGTTTTGG | *floT* rv (KpnI) |
| JS105 | TTTTGCATGCATGGATCCGTCAACACTTATG | *floA* fw (SphI) |
| JS106 | TTTTGGTACCCGTGATTTGCGGTCTTCATCCG | *floA* rv (KpnI) |
| JS107 | TTTTGCATGCATGAAATTTTGGAAAAGCGTAG | *resE* fw (SphI) |
| JS108 | TTTTGGTACCCGCCGTTTTGTCGGAATATAAAAAG | *resE* rv (KpnI) |
| JS109 | TTTTGCATGCATGAATAAATACCGTGTGCGCC | *phoR* fw (SphI) |
| JS110 | TTTTGGTACCCGGGCGGACTTTTCAGCGGCC | *phoR* rv (KpnI) |
| JS130 | TTTTGCATGCTTACTATTTATCGTCGTCATCTTTG | *flag* rv (SphI) |
| JS126 | GGAGGAGCCAAAAATGAATGAAGACTACAAAGACCATGACGGTG | *flag* fw (PCR *phoP*) |
| JS127 | CACCGTCATGGTCTTTGTAGTCTTCATTCATTTTTGGCTCCTCC | *phoP* rv (PCR *flag*) |
| JS128 | TTATAAATTTGAGGTCGGCGCTGAAGACTACAAAGACCATGACGGTG | *flag* fw (PCR *resD*) |
| JS129 | CACCGTCATGGTCTTTGTAGTCTTCAGCGCCGACCTCAAATTTATAA | *resD* rv (PCR *flag*) |
| JS131 | AAAAGTCGACTAAGGAGGAACTACTATGAACAAGAAAATTTTAGTTGTGG | *phoP* fw (SalI) |
| JS132 | AAAAGTCGACTAAGGAGGAACTACTATGGACCAAACGAACGAAACAAA | *resD* fw (SalI) |
| JS133 | GCGAAGAATTTCTTTTTCTTCTAGAATATCAATTGAGAGAATTTCAAAC | *floA* N-terminus rv (PCR *floA_T_*) |
| JS134 | GTTTGAAATTCTCTCAATTGATATTCTAGAAGAAAAAGAAATTCTTCGC | *floT* C-terminus fw (PCR *floA_T_*) |
| JS135 | TTTTTGCCGATATCTACATCTGCTTCTATTTGTTTTTGGCGTTCGAT | *floT* N-terminus rv (PCR *floT_A_*) |
| JS136 | ATCGAACGCCAAAAACAAATAGAAGCAGATGTAGATATCGGCAAAAA | *floA* C-terminus fw (PCR *floT_A_*) |
| JS137 | GTCGGCGCTGAATGAAATTTTGCAGCGAACCATTTGAGGTGATA | *km* fw (PCR *resE* up) |
| JS138 | TATCACCTCAAATGGTTCGCTGCAAAATTTCATTCAGCGCCGAC | *resE* up rv (PCR *km*) |
| JS139 | GCCTACGAGGAATTTGTATCGCGGTAAAATCGAGTCTGAATTTG | *resE* down fw (PCR *km*) |
| JS140 | CAAATTCAGACTCGATTTTACCGCGATACAAATTCCTCGTAGGC | *km* rv (PCR *resE* down) |
| JS141 | TTTTGTCGACCGACACCCATTATTATGCTGAC | *resE* up fw (SalI) |
| JS142 | TTTTGCATGCCGGCAGCAATTGCTGATCCC | *resE* down rv (SphI) |
| JS143 | CTGGAGGAGCCAAAAATGAATGCAGCGAACCATTTGAGGTGATA | *km* fw (PCR *phoR* up) |
| JS144 | TATCACCTCAAATGGTTCGCTGCATTCATTTTTGGCTCCTCCAG | *phoR* up rv (PCR *km*) |
| JS145 | GCCTACGAGGAATTTGTATCGGTCCGCCTAATGTTTACAAAGG | *phoR* down fw (PCR *km*) |
| JS146 | CCTTTGTAAACATTAGGCGGACCGATACAAATTCCTCGTAGGC | *km* rv (PCR *phoR* down) |
| JS147 | TTTTGTCGACCCCATTTTAATGCTGACAGCG | *phoR* up fw (SalI) |
| JS148 | TTTTGCATGCCAGGCGGGGTTTTCTGTCTG | *phoR* down rv (SphI) |
| oTB42 | AGGATTGGAAGCTGTTCGT | *girB* fw |
| oTB43 | TGACTTCTACCGCAGGAC | *girB* rv |
| oTB44 | CAAAGGTTGTGCAACATGC | *sboAX* |
| oTB45 | CCCATAGACCGAATAGACCT | *sboAX* rv |
| oTB46 | TGTCTTTCGTCCATTCGCT | *albC* fw |
| oTB47 | GATTCCACATCCAAACCGAC | *albC* rv |
| oTB48 | TAGCGATGAGGAATGGGAGG | *dhbA* fw |
| oTB49 | CGTAAACATCACAGCCGCA | *dhbA* rv |
| oTB50 | ATATCGCCGTGACCCTC | *argC* fw |
| oTB51 | CCAGTTTCTTCATTCCAGCC | *argC* rv |
| oTB52 | GGCAGTCTAATTCCTCCGT | *cydB* fw |
| oTB53 | CAAAGAAGCGTTACCGTCAC | *cydB* rv |
| oTB54 | GCATTGAACAGATTGAGGGA | *pyrR* fw |
| oTB55 | CATCCATTCCTGCTCTGAC | *pyrR* rv |
| oTB94 | GATTCTGTCTTACGAAACCGCT | *glpQ* fw |
| oTB95 | GTCTTTCACCCAACCCATTCC | *glpQ* rv |
