# Supplementary material for: Spatio-temporal Remodeling of Functional Membrane Microdomains Organizes the Signaling Networks of a Bacterium
Source: PLoS Genet. 2015 Apr 24;11(4):e1005140. doi: 10.1371/journal.pgen.1005140 (PMC4409396; doi:10.1371/journal.pgen.1005140)
Supplement: S3 Table — (DOCX) [file pgen.1005140.s009.docx]

**Supplemental Table S3 (Related to main figure 6):** List of proteins identified in membrane-associated protein complexes that interacted exclusively with either FloA () or FloT (), or FloA and FloT ().

|  | **Protein** | **Description** | **Functional category** | **sequence coverage FloA / FloT** | |
| --- | --- | --- | --- | --- | --- |
|  | Eno | enolase, glycolytic/ gluconeogenic enzyme | Post-exponential lifestyles | 60% | 55% |
|  | FtsH*^†^ | cell-division protein / general stress protein (class III heat-shock) |  | 28% | 30% |
|  | McpA^†^ | methyl-accepting chemotaxis protein |  | - | 25% |
|  | McpB^†^ | methyl-accepting chemotaxis protein |  | - | 21% |
|  | Rny | RNase Y, 5' end sensitive endoribonuclease, involved in the degradation/processing of mRNA |  | 40% | 31% |
|  | YcdA* | lipoprotein, required for swarming motility |  | - | 60% |
|  | MreC* | cell-shape determining protein | Cell envelope/ Cell divison | 41% | - |
|  | PhoR | two-component sensor histidine kinase involved in phosphate regulation |  | 22% | - |
|  | PonA | penicillin-binding proteins 1A/1B |  | 23% | - |
|  | RasP | control of cell division and SigW activity |  | 26% | 29% |
|  | RodZ | required for cell shape determination |  | 31% | 40% |
|  | TagU^†^ | protein involved in cell wall teichoic acid biosynthesis |  | 33% | - |
|  | YceH | unknown; similar to toxic anion resistance protein | Coping with stress | 41% | - |
|  | YtxH | unknown; similar to general stress protein |  | 60% | - |
|  | FloA^†^ | flottilin-like protein (in addition to FloT), resistence protein (against sublancin) | Membrane dynamics | 48% | 51% |
|  | FloT* | similar to flotillin 1, orchestration of physiological processes in lipid microdomains |  | - | 81% |
|  | AtpA* | ATP synthase (subunit alpha) | Metabolism | 56% | 55% |
|  | AtpB* | ATP synthase (subunit a) |  | 86% | 75% |
|  | AtpG^†^ | ATP synthase (subunit gamma) |  | 59% | 40% |
|  | DhaS | aldehyde dehydrogenase |  | 40% | - |
|  | MsmE | multiple sugar-binding protein |  | 61% | - |
|  | QoxA* | cytochrome aa3 quinol oxidase (subunit II) |  | 52% | 62% |
|  | RocG | arginine utilization, controls the activity of GltC |  | 51% | 54% |
|  | SdhA^†^ | succinate dehydrogenase (flavoprotein subunit) |  | 39% | 44% |
|  | BdbD* | thiol-disulfide oxidoreductase | Protein secretion/ modification | 71% | 73% |
|  | PrkC | protein kinase C, induce germination of spores in response to DAP-type, and not to Lys-type cell wall muropeptides |  | - | 49% |
|  | PrsA* | protein secretion (post-translocation molecular chaperone) |  | 84% | 83% |
|  | ResE | two-component sensor kinase, regulation of aerobic and anaerobic respiration |  | - | 30% |
|  | SecA | preprotein translocase subunit (ATPase) |  | - | 62% |
|  | SecDF | protein-export membrane protein |  | - | 38% |
|  | SpoIIIAH | component of the SpoIIIA-SpoIIQ type III secretion system residing in the forespore membrane |  | 46% | 37% |
|  | SpoIIQ | component of the SpoIIIAH-SpoIIQ type III secretion system residing in the forespore membrane |  | 82% | 66% |
|  | YacD | similar to secretion protein PrsA |  | - | 52% |
|  | AapA | amino acid permease | Transport-Homeostasis | 51% | - |
|  | AppA | oligopeptide ABC transporter (oligopeptide-binding protein) |  | 60% | 69% |
|  | FeuA* | ABC transporter for the siderophores Fe-enterobactin and Fe-bacillibactin |  | 81% | 68% |
|  | FhuD*^†^ | ferrichrome ABC transporter (ferrichrome-binding protein) |  | 32% | 48% |
|  | MntA^†^ | manganese ABC transporter (membrane protein) |  | 62% | 76% |
|  | MntB | manganese ABC transporter (ATP-binding protein) |  | 64% | 52% |
|  | NupO | ABC transporter for guanosine (ATP-binding protein) |  | - | 39% |
|  | OppA*^†^ | oligopeptide ABC transporter (binding protein) (initiation of sporulation, competence development) |  | 53% | 78% |
|  | OppD | oligopeptide ABC transporter (ATP-binding protein) (initiation of sporulation, competence development) |  | 58% | 69% |
|  | OppF | oligopeptide ABC transporter (ATP-binding protein) (initiation of sporulation, competence development) |  | 37% | 63% |
|  | OpuAA | glycine betaine ABC transporter (ATP-binding protein) |  | 52% | 40% |
|  | YclQ^†^ | petrobactin (3.4-catecholate siderophore) ABC transporter (binding protein) |  | - | 77% |
|  | YfiY^†^ | ABC transporter for the siderophore schizokinen and arthrobactin |  | - | 75% |
|  | YfmC | iron/ citrate ABC transporter (binding protein) |  | 62% | 56% |
|  | YhfQ^†^ | iron/ citrate ABC transporter (solute-binding protein) |  | - | 37% |
|  | YknX | ABC-type antimicrobial peptide transporter (permease) for the export of the SdpC toxin |  | - | 60% |
|  | YxeB* | hydroxamate siderophore ABC transporter (only ferrioxamine) |  | 60% | 53% |
|  | yxeM* | putative cysteine ABC transporter (binding protein) |  | - | 78% |
|  | RnjA | RNase J1 | RNA synthesis /degradation | - | 43% |
