# Supplementary material for: Spatio-temporal Remodeling of Functional Membrane Microdomains Organizes the Signaling Networks of a Bacterium
Source: PLoS Genet. 2015 Apr 24;11(4):e1005140. doi: 10.1371/journal.pgen.1005140 (PMC4409396; doi:10.1371/journal.pgen.1005140)
Supplement: S4 Table — Mean indicates log 2 transformed expression ratios. (DOCX) [file pgen.1005140.s010.docx]

**Supplemental Table S4 (Related to main figures 7 and 8):** List of genes that are significantly up or downregulated (Bayes.p value <10-4) in the Δ*floA* cells compared to wild-type cells. Mean indicates log 2 transformed expression ratios.

| locus tag | gene | Mean | Bayes.p | annotation |
| --- | --- | --- | --- | --- |
| BSU25380 | *floA* | -1,62 | 10^-9^ | flotillin-like protein |
| BSU15510 | *pyrAA* | -1,21 | 10^-7^ | carbamoyl phosphate synthase small subunit |
| BSU40660 | *yybF* | -1,11 | 10^-6^ | permease |
| BSU11220 | *argD* | -1,07 | 10^-7^ | acetylornithine aminotransferase |
| BSU11230 | *carA* | -1,02 | 10^-6^ | carbamoyl phosphate synthase small subunit |
| BSU15520 | *pyrAB* | -0,96 | 10^-7^ | carbamoyl phosphate synthase large subunit |
| BSU11210 | *argB* | -0,93 | 10^-6^ | acetylglutamate kinase |
| BSU29440 | *argH* | -0,91 | 10^-6^ | argininosuccinate lyase |
| BSU11190 | *argC* | -0,85 | 10^-6^ | N-acetyl-gamma-glutamyl-phosphate reductase |
| BSU11250 | *argF* | -0,84 | 10^-6^ | ornithine carbamoyltransferase |
| BSU29450 | *argG* | -0,83 | 10^-5^ | argininosuccinate synthase |
| BSU10410 | *yhzC* | -0,78 | 10^-6^ | hypothetical protein |
| BSU11200 | *argJ* | -0,78 | 10^-4^ | ornithine acetyltransferase |
| BSU38750 | *cydB* | -0,77 | 10^-4^ | cytochrome bd ubiquinol oxidase subunit II |
| BSU27330 | *udk* | -0,76 | 10^-4^ | uridine kinase |
| BSU15530 | *pyrK* | -0,74 | 10^-5^ | dihydroorotate dehydrogenase electron transfer subunit |
| BSU23980 | *yqiX* | -0,71 | 10^-5^ | high affinity arginine ABC transporter binding lipoprotein |
| BSU13750 | *ykvM* | -0,70 | 10^-5^ | 7-cyano-7-deazaguanine reductase |
| BSU03060 | *lctP* | -0,68 | 10^-4^ | L-lactate permease |
| BSU19550 | *yodC* | -0,64 | 10^-4^ | oxidoreductase |
| BSU23960 | *yqiZ* | -0,62 | 10^-5^ | high affinity arginine ABC transporter ATP-binding protein |
| BSU00140 | *dck* | -0,59 | 10^-4^ | deoxyadenosine/deoxycytidine kinase |
| BSU38570 | *licA* | -0,57 | 10^-4^ | PTS system lichenan-specific transporter subunit IIA |
| BSU13740 | *ykvL* | -0,53 | 10^-4^ | queuosine biosynthesis enzyme |
| BSU16580 | *polC* | -0,53 | 10^-4^ | DNA polymerase III |
| BSU02130 | *glpQ* | -0,53 | 10^-4^ | glycerophosphoryl diester phosphodiesterase |
| BSU06170 | *ydjE* | -0,52 | 10^-4^ | sugar kinase |
| BSU09460 | *yhdG* | -0,52 | 10^-4^ | branched-chain amino acid transporter |
| BSU17400 | *ymaB* | -0,51 | 10^-4^ | hypothetical protein |
| BSU11760 | *cotX* | -0,51 | 10^-4^ | spore coat protein |
| BSU14590 | *pdhB* | -0,51 | 10^-4^ | pyruvate dehydrogenase E1 component subunit beta |
| BSU11700 | *thiF* | -0,49 | 10^-4^ | thiamine/molybdopterin biosynthesis |
| BSU04200 | *ydaE* | -0,48 | 10^-4^ | hypothetical protein |
| BSU18450 | *gltA* | -0,47 | 10^-4^ | glutamate synthase large subunit |
| BSU15210 | *spoVE* | -0,45 | 10^-4^ | factor for spore cortex peptidoglycan synthesis |
| BSU35730 | *tagE* | 0,37 | 10^-4^ | alpha-glucosyltransferase |
| BSU35700 | *tagH* | 0,40 | 10^-4^ | teichoic acid transport system ATP-binding protein |
| BSU35710 | *tagG* | 0,42 | 10^-4^ | teichoic acid translocation permease protein |
| BSU28040 | *radC* | 0,42 | 10^-4^ | DNA repair protein |
| BSU08690 | *ygaD* | 0,43 | 10^-4^ | ABC transporter ATP-binding protein |
| BSU35750 | *tagA* | 0,43 | 10^-4^ | N-acetylmannosaminyltransferase |
| BSU20880 | *yopI* | 0,45 | 10^-4^ | hypothetical protein |
| BSU02860 | *ycdI* | 0,46 | 10^-4^ | Zn(II) transporter ATP-binding protein |
| BSU13270 | *ykoI* | 0,46 | 10^-4^ | hypothetical protein |
| BSU37160 | *rpoE* | 0,46 | 10^-4^ | DNA-directed RNA polymerase subunit delta |
| BSU19390 | *yojN* | 0,47 | 10^-4^ | nitric-oxide reductase |
| BSU17350 | *ymzC* | 0,47 | 10^-4^ | hypothetical protein |
| BSU22910 | *ypfA* | 0,48 | 10^-4^ | cyclic diGMP binding protein |
| BSU32870 | *yusO* | 0,48 | 10^-4^ | MarR family transcriptional regulator |
| BSU20950 | *yopB* | 0,49 | 10^-4^ | transcriptional regulator |
| BSU05050 | *lrpA* | 0,50 | 10^-4^ | Lrp/AsnC family transcriptional regulator |
| BSU35760 | *tagB* | 0,50 | 10^-4^ | CDP-glycerol:glycerophosphate glycerophosphotransferase |
| BSU00270 | *yaaO* | 0,51 | 10^-4^ | lysine decarboxylase |
| BSU31890 | *yukC* | 0,51 | 10^-5^ | bacteriocin production protein |
| BSU06400 | *yebE* | 0,52 | 10^-4^ | hypothetical protein |
| BSU39910 | *yxnB* | 0,52 | 10^-4^ | hypothetical protein |
| BSU37210 | *ywjC* | 0,52 | 10^-4^ | hypothetical protein |
| BSU20840 | *yopM* | 0,53 | 10^-4^ | hypothetical protein |
| BSU01780 | *glmS* | 0,53 | 10^-4^ | glucosamine--fructose-6-phosphate aminotransferase |
| BSU00010 | *dnaA* | 0,53 | 10^-4^ | chromosome replication initiator |
| BSU40180 | *yydF* | 0,54 | 10^-4^ | peptide controlling LiaRS |
| BSU35720 | *tagF* | 0,54 | 10^-4^ | CDP-glycerol glycerophosphotransferase |
| BSU00710 | *yacC* | 0,55 | 10^-4^ | heat shock protein |
| BSU25680 | *yqeG* | 0,56 | 10^-5^ | hydrolase |
| BSU14960 | *ylbC* | 0,56 | 10^-4^ | hypothetical protein |
| BSU09410 | *phoA* | 0,56 | 10^-4^ | alkaline phosphatase |
| BSU25720 | *yqeD* | 0,56 | 10^-4^ | hypothetical protein |
| BSU01750 | *ybbP* | 0,57 | 10^-4^ | hypothetical protein |
| BSU00750 | *pabA* | 0,57 | 10^-4^ | anthranilate synthase glutamine amidotransferase |
| BSU16120 | *topA* | 0,57 | 10^-4^ | DNA topoisomerase I |
| BSU11640 | *yjbQ* | 0,57 | 10^-4^ | Na+/H+ antiporter |
| BSU15120 | *yllA* | 0,57 | 10^-4^ | nucleoid associated protein |
| BSU20790 | *yopR* | 0,57 | 10^-4^ | integrase |
| BSU20800 | *yopQ* | 0,57 | 10^-4^ | hypothetical protein |
| BSU27520 | *yrzC* | 0,57 | 10^-4^ | cysteine biosynthesis transcriptional regulator |
| BSU16950 | *pbpX* | 0,57 | 10^-4^ | penicillin-binding endopeptidase X |
| BSU15300 | *bpr* | 0,58 | 10^-4^ | bacillopeptidase F |
| BSU00100 | *dacA* | 0,58 | 10^-4^ | D-alanyl-D-alanine carboxypeptidase |
| BSU41020 | *thdF* | 0,58 | 10^-4^ | tRNA modification GTPase |
| BSU03300 | *nasD* | 0,59 | 10^-5^ | assimilatory nitrite reductase subunit |
| BSU20910 | *yopF* | 0,59 | 10^-4^ | hypothetical protein |
| BSU14170 | *ykuP* | 0,59 | 10^-4^ | short-chain flavodoxin |
| BSU21730 | *ypmS* | 0,60 | 10^-4^ | hypothetical protein |
| BSU37430 | *albG* | 0,60 | 10^-4^ | hypothetical protein |
| BSU10980 | *yitG* | 0,61 | 10^-4^ | efflux transporter |
| BSU40440 | *dnaC* | 0,61 | 10^-4^ | replicative DNA helicase |
| BSU40610 | *yybK* | 0,62 | 10^-4^ | hypothetical protein |
| BSU37380 | *albB* | 0,62 | 10^-4^ | hypothetical protein |
| BSU28600 | *yshB* | 0,62 | 10^-5^ | hypothetical protein |
| BSU09330 | *yhcZ* | 0,63 | 10^-4^ | two-component response regulator |
| BSU05470 | *ydfM* | 0,65 | 10^-4^ | divalent cation efflux transporter |
| BSU25140 | *yqfR* | 0,65 | 10^-5^ | ATP-dependent RNA helicase |
| BSU01960 | *ybdB* | 0,65 | 10^-5^ | sporulation killing factor biosynthesis and export |
| BSU23210 | *ypuH* | 0,67 | 10^-5^ | chromosome condensation and segregation factor |
| BSU13040 | *hmp* | 0,67 | 10^-4^ | nitric oxide dioxygenase |
| BSU02870 | *yceA* | 0,69 | 10^-5^ | high affinity Zn(II) ABC transporter permease |
| BSU14630 | *speA* | 0,70 | 10^-5^ | arginine decarboxylase |
| BSU30430 | *ytrD* | 0,70 | 10^-4^ | ABC transporter permease |
| BSU30050 | *ytgP* | 0,70 | 10^-4^ | hypothetical protein |
| BSU35260 | *ftsE* | 0,71 | 10^-5^ | cell-division ABC transporter ATP-binding protein |
| BSU09510 | *yhdL* | 0,72 | 10^-5^ | negative regulator of the activity of sigmaM |
| BSU08600 | *csbB* | 0,75 | 10^-6^ | glycosyl transferase family protein |
| BSU21130 | *yonD* | 0,75 | 10^-5^ | hypothetical protein |
| BSU13060 | *ykjA* | 0,76 | 10^-5^ | hypothetical protein |
| BSU39920 | *asnH* | 0,76 | 10^-6^ | asparagine synthetase |
| BSU17370 | *ymaA* | 0,76 | 10^-5^ | ribonucleotide reductase stimulatory protein |
| BSU21050 | *yonN* | 0,76 | 10^-5^ | HU-related DNA-binding protein |
| BSU15490 | *pyrB* | 0,77 | 10^-4^ | aspartate carbamoyltransferase |
| BSU00800 | *yazB* | 0,79 | 10^-6^ | transcriptional regulator |
| BSU09520 | *sigM* | 0,81 | 10^-6^ | RNA polymerase sigma factor |
| BSU00780 | *folB* | 0,83 | 10^-5^ | dihydroneopterin aldolase |
| BSU19160 | *yocC* | 0,84 | 10^-6^ | hypothetical protein |
| BSU30450 | *ytrB* | 0,86 | 10^-5^ | ABC transporter ATP-binding protein |
| BSU00770 | *sul* | 0,90 | 10^-6^ | dihydropteroate synthase |
| BSU23220 | *ypuG* | 0,90 | 10^-7^ | segregation and condensation protein A |
| BSU13300 | *ykoK* | 0,90 | 10^-6^ | magnesium transporter |
| BSU21090 | *yonH* | 0,95 | 10^-6^ | capsid protein |
| BSU37400 | *albD* | 0,96 | 10^-8^ | hypothetical protein |
| BSU00790 | *folK* | 0,98 | 10^-6^ | 7, 8-dihydro-6-hydroxymethylpterin-pyrophosphokinase |
| BSU37410 | *albE* | 1,00 | 10^-8^ | hydrolase |
| BSU37420 | *albF* | 1,00 | 10^-7^ | subtilosin production peptidase |
| BSU05490 | *ydfO* | 1,01 | 10^-5^ | dioxygenase |
| BSU37390 | *albC* | 1,02 | 10^-6^ | subtilosin production transporter |
| BSU00760 | *pabC* | 1,05 | 10^-7^ | 4-amino-4-deoxychorismate lyase |
| BSU15480 | *pyrP* | 1,10 | 10^-6^ | uracil permease |
| BSU30440 | *ytrC* | 1,11 | 10^-7^ | ABC transporter permease |
| BSU30460 | *ytrA* | 1,12 | 10^-7^ | GntR family transcriptional regulator |
| BSU15470 | *pyrR* | 1,52 | 10^-8^ | bifunctional pyrimidine regulatory protein |
|  |  |  |  |  |
