# Supplementary material for: Spatio-temporal Remodeling of Functional Membrane Microdomains Organizes the Signaling Networks of a Bacterium
Source: PLoS Genet. 2015 Apr 24;11(4):e1005140. doi: 10.1371/journal.pgen.1005140 (PMC4409396; doi:10.1371/journal.pgen.1005140)
Supplement: S5 Table — Mean indicates log 2 transformed expression ratios. (DOCX) [file pgen.1005140.s011.docx]

**Supplemental Table S5 (Related to main figures 7 and 8):** List of genes that are significantly up or downregulated (Bayes.p value <10-4) in the Δ*floT* cells compared to wild-type cells. Mean indicates log 2 transformed expression ratios.

| locus tag | gene | Mean | Bayes.p | annotation |
| --- | --- | --- | --- | --- |
| BSU31010 | *floT* | -1,89 | 10^-9^ | flotillin-like protein |
| BSU03300 | *nasD* | -1,04 | 10^-8^ | assimilatory nitrite reductase subunit |
| BSU13040 | *hmp* | -1,00 | 10^-7^ | nitric oxide dioxygenase |
| BSU31990 | *dhbC* | -0,93 | 10^-6^ | isochorismate synthase |
| BSU14150 | *ykuN* | -0,93 | 10^-9^ | flavodoxin |
| BSU31980 | *dhbE* | -0,92 | 10^-7^ | 2,3-dihydroxybenzoate-AMP ligase |
| BSU37360 | *sboX* | -0,92 | 10^-7^ | bacteriocin-like product |
| BSU17660 | *yncF* | -0,92 | 10^-4^ | deoxyuridine 5'-triphosphate pyrophosphatase |
| BSU14160 | *ykuO* | -0,88 | 10^-9^ | hypothetical protein |
| BSU37350 | *sboA* | -0,84 | 10^-7^ | subtilosin A |
| BSU22550 | *qcrB* | -0,83 | 10^-5^ | cytochrome b |
| BSU31970 | *dhbB* | -0,83 | 10^-6^ | isochorismatase |
| BSU32000 | *dhbA* | -0,83 | 10^-6^ | 2,3-dihydroxybenzoate-2,3-dehydrogenase |
| BSU11990 | *yjdB* | -0,81 | 10^-4^ | hypothetical protein |
| BSU31960 | *dhbF* | -0,79 | 10^-7^ | siderophore bacillibactin synthetase |
| BSU04530 | *ydbN* | -0,77 | 10^-6^ | hypothetical protein |
| BSU38750 | *cydB* | -0,72 | 10^-4^ | cytochrome bd ubiquinol oxidase subunit II |
| BSU19680 | *yozE* | -0,70 | 10^-4^ | hypothetical protein |
| BSU20580 | *yoqM* | -0,69 | 10^-6^ | membrane bound protein |
| BSU37390 | *albC* | -0,69 | 10^-4^ | subtilosin production transporter |
| BSU16620 | *ylxQ* | -0,69 | 10^-4^ | hypothetical protein |
| BSU30020 | *ytzE* | -0,67 | 10^-4^ | DeoR family transcriptional regulator |
| BSU37310 | *fnr* | -0,65 | 10^-6^ | FNR/CAP family transcriptional regulator |
| BSU32010 | *besA* | -0,61 | 10^-4^ | bacillibactin trilactone hydrolase |
| BSU04810 | *immA* | -0,51 | 10^-4^ | immunity anti-repressor |
| BSU11010 | *yitJ* | -0,51 | 10^-4^ | homocysteine S-methyltransferase |
| BSU18380 | *iseA* | -0,51 | 10^-6^ | inhibitor of cell-separation enzymes |
| BSU11360 | *appD* | -0,47 | 10^-5^ | oligopeptide ABC transporter ATP-binding protein |
| BSU37370 | *albA* | -0,47 | 10^-5^ | antilisterial bacteriocin (subtilosin) production protein |
| BSU00370 | *abrB* | -0,47 | 10^-4^ | transcriptional regulator |
| BSU40180 | *yydF* | -0,46 | 10^-5^ | peptide controlling LiaRS |
| BSU03230 | *ycgP* | -0,46 | 10^-4^ | transcriptional regulator |
| BSU24620 | *tasA* | -0,46 | 10^-4^ | major biofilm matrix component |
| BSU17710 | *tatAC* | -0,46 | 10^-4^ | twin-arginine pre-protein translocation pathway protein |
| BSU19300 | *yozC* | -0,43 | 10^-4^ | hypothetical protein |
| BSU02040 | *ybdN* | -0,43 | 10^-4^ | hypothetical protein |
| BSU33770 | *spbC* | -0,43 | 10^-4^ | sporulation killing factor |
| BSU20420 | *yorD* | -0,42 | 10^-4^ | hypothetical protein |
| BSU05030 | *yddM* | -0,41 | 10^-5^ | helicase |
| BSU04820 | *immR* | -0,41 | 10^-4^ | XRE family transcriptional regulator |
| BSU01620 | *feuB* | -0,38 | 10^-4^ | iron-uptake protein |
| BSU31030 | *yuaE* | -0,38 | 10^-4^ | hypothetical protein |
| BSU33750 | *sdpA* | -0,38 | 10^-4^ | export of killing factor |
| BSU05020 | *phrI* | -0,38 | 10^-4^ | secreted regulator of the activity of phosphatase RapI |
| BSU03780 | *phrC* | -0,35 | 10^-4^ | secreted regulator of the activity of phosphatase RapC |
| BSU23150 | *resA* | -0,34 | 10^-4^ | thiol-disulfide oxidoreductase |
| BSU33600 | *smpB* | -0,34 | 10^-4^ | SsrA-binding protein |
| BSU00490 | *spoVG* | -0,33 | 10^-4^ | regulatory protein |
| BSU18310 | *ppsD* | -0,32 | 10^-4^ | plipastatin synthetase |
| BSU35750 | *tagA* | -0,24 | 10^-4^ | N-acetylmannosaminyltransferase |
| BSU31580 | *maeN* | 0,31 | 10^-4^ | Na+/malate symporter |
| BSU01780 | *glmS* | 0,35 | 10^-4^ | glucosamine--fructose-6-phosphate aminotransferase |
| BSU21810 | *dfrA* | 0,36 | 10^-4^ | dihydrofolate reductase |
| BSU29600 | *braB* | 0,37 | 10^-4^ | branched-chain amino acid/Na+ symporter |
| BSU14030 | *ykuC* | 0,37 | 10^-4^ | efflux transporter |
| BSU09280 | *glpF* | 0,39 | 10^-4^ | glycerol permease |
| BSU37150 | *pyrG* | 0,39 | 10^-4^ | CTP synthetase |
| BSU06430 | *purK* | 0,41 | 10^-4^ | phosphoribosylaminoimidazole carboxylase ATPase subunit |
| BSU16950 | *pbpX* | 0,41 | 10^-4^ | penicillin-binding endopeptidase |
| BSU07340 | *yfnA* | 0,41 | 10^-4^ | metabolite permease |
| BSU03580 | *yczE* | 0,45 | 10^-4^ | integral inner membrane protein |
| BSU03050 | *ldh* | 0,48 | 10^-4^ | L-lactate dehydrogenase |
| BSU15490 | *pyrB* | 0,48 | 10^-4^ | aspartate carbamoyltransferase |
| BSU23980 | *artP* | 0,50 | 10^-4^ | high affinity arginine ABC transporter binding lipoprotein |
| BSU23970 | *artQ* | 0,58 | 10^-6^ | high affinity arginine ABC transporter permease |
| BSU29450 | *argG* | 0,60 | 10^-5^ | argininosuccinate synthase |
| BSU15470 | *pyrR* | 0,64 | 10^-4^ | bifunctional pyrimidine regulatory protein PyrR/uracil phosphoribosyltransferase |
| BSU33330 | *lysP* | 0,66 | 10^-5^ | lysine permease |
