# Supplementary material for: Spatio-temporal Remodeling of Functional Membrane Microdomains Organizes the Signaling Networks of a Bacterium
Source: PLoS Genet. 2015 Apr 24;11(4):e1005140. doi: 10.1371/journal.pgen.1005140 (PMC4409396; doi:10.1371/journal.pgen.1005140)
Supplement: S6 Table — Mean indicates log 2 transformed expression ratios. (DOCX) [file pgen.1005140.s012.docx]

**Supplemental Table S6 (Related to main figures 7 and 8):** List of genes that are significantly up or downregulated (Bayes.p value <10-4) in the Δ*floA* Δ*floT* cells compared to wild-type cells. Mean indicates log 2 transformed expression ratios.

| **locus tag** | **gene** | **Mean** | **Bayes.p** | **annotation** |
| --- | --- | --- | --- | --- |
| BSU31010 | *floT* | -1,77 | 10^-7^ | flotillin-like protein |
| BSU36750 | *spoIID* | -1,49 | 10^-7^ | stage II sporulation autolysin |
| BSU14150 | *ykuN* | -1,30 | 10^-6^ | flavodoxin |
| BSU13750 | *ykvM* | -1,28 | 10^-5^ | 7-cyano-7-deazaguanine reductase |
| BSU14160 | *ykuO* | -1,21 | 10^-6^ | hypothetical protein |
| BSU13740 | *ykvL* | -1,18 | 10^-6^ | queuosine biosynthesis enzyme |
| BSU31970 | *dhbB* | -1,10 | 10^-5^ | isochorismatase |
| BSU37360 | *sboX* | -1,09 | 10^-6^ | bacteriocin-like product |
| BSU25380 | *floA* | -1,08 | 10^-5^ | flotillin-like protein |
| BSU31980 | *dhbE* | -1,07 | 10^-5^ | 2,3-dihydroxybenzoate-AMP ligase |
| BSU31960 | *dhbF* | -1,07 | 10^-4^ | bacillibactin synthetase |
| BSU18450 | *gltA* | -1,05 | 10^-6^ | glutamate synthase large subunit |
| BSU13730 | *ykvK* | -1,04 | 10^-5^ | 6-pyruvoyl tetrahydrobiopterin synthase |
| BSU03300 | *nasD* | -1,03 | 10^-5^ | assimilatory nitrite reductase subunit |
| BSU13920 | *splA* | -1,00 | 10^-4^ | TRAP-like transcriptional regulator |
| BSU37350 | *sboA* | -0,96 | 10^-5^ | subtilosin A |
| BSU31990 | *dhbC* | -0,95 | 10^-4^ | isochorismate synthase |
| BSU04530 | *ydbN* | -0,93 | 10^-5^ | hypothetical protein |
| BSU17900 | *yneE* | -0,92 | 10^-4^ | hypothetical protein |
| BSU13720 | *ykvJ* | -0,91 | 10^-4^ | pre-queuosine 0 synthase |
| BSU40660 | *yybF* | -0,91 | 10^-4^ | permease |
| BSU38750 | *cydB* | -0,88 | 10^-4^ | cytochrome bd ubiquinol oxidase subunit II |
| BSU18560 | *yoaD* | -0,86 | 10^-4^ | 2-hydroxyacid dehydrogenase |
| BSU11010 | *yitJ* | -0,82 | 10^-4^ | homocysteine S-methyltransferase |
| BSU18440 | *gltB* | -0,81 | 10^-4^ | glutamate synthase subunit beta |
| BSU32720 | *yurZ* | -0,81 | 10^-5^ | hypothetical protein |
| BSU10410 | *yhzC* | -0,78 | 10^-4^ | hypothetical protein |
| BSU11000 | *yitI* | -0,78 | 10^-4^ | N-acetyltransferase |
| BSU11360 | *appD* | -0,77 | 10^-5^ | oligopeptide ABC transporter ATP-binding protein |
| BSU01140 | *ybaC* | -0,76 | 10^-4^ | proline iminopeptidase |
| BSU37380 | *albB* | -0,74 | 10^-4^ | hypothetical protein |
| BSU19370 | *odhA* | -0,74 | 10^-4^ | 2-oxoglutarate dehydrogenase |
| BSU06850 | *yeeK* | -0,62 | 10^-4^ | spore associated protein |
| BSU03010 | *amhX* | -0,54 | 10^-4^ | amidohydrolase |
| BSU01120 | *fusA* | -0,52 | 10^-4^ | elongation factor G |
| BSU08860 | *ssuD* | -0,50 | 10^-4^ | alkanesulfonate monooxygenase |
| BSU35700 | *tagH* | 0,58 | 10^-4^ | teichoic acid transport system ATP-binding protein |
| BSU06420 | *purE* | 0,58 | 10^-4^ | phosphoribosylaminoimidazole carboxylase I |
| BSU24940 | *yqzC* | 0,60 | 10^-4^ | hypothetical protein |
| BSU29440 | *argH* | 0,60 | 10^-4^ | argininosuccinate lyase |
| BSU15500 | *pyrC* | 0,61 | 10^-4^ | dihydroorotase |
| BSU03850 | *ycnC* | 0,62 | 10^-4^ | TetR family transcriptional regulator |
| BSU19210 | *yocH* | 0,64 | 10^-4^ | cell wall-binding protein |
| BSU31890 | *yukC* | 0,65 | 10^-4^ | bacteriocin production protein |
| BSU25140 | *yqfR* | 0,66 | 10^-4^ | ATP-dependent RNA helicase; cold shock |
| BSU29600 | *braB* | 0,67 | 10^-4^ | branched-chain amino acid/Na^+^ symporter |
| BSU23960 | *yqiZ* | 0,67 | 10^-4^ | high affinity arginine ABC transporter ATP-binding protein |
| BSU40100 | *ahpF* | 0,68 | 10^-4^ | alkyl hydroperoxide reductase |
| BSU18120 | *alsT* | 0,68 | 10^-4^ | amino acid carrier protein |
| BSU29570 | *sspA* | 0,69 | 10^-4^ | small acid-soluble spore protein |
| BSU06430 | *purK* | 0,69 | 10^-4^ | phosphoribosylaminoimidazole carboxylase ATPase subunit |
| BSU09710 | *yheI* | 0,70 | 10^-4^ | ABC transporter ATP-binding protein |
| BSU23210 | *ypuH* | 0,70 | 10^-4^ | chromosome condensation and segregation factor |
| BSU37150 | *pyrG* | 0,70 | 10^-4^ | CTP synthetase |
| BSU03580 | *yczE* | 0,71 | 10^-4^ | integral inner membrane protein |
| BSU11210 | *argB* | 0,72 | 10^-4^ | acetylglutamate kinase |
| BSU00010 | *dnaA* | 0,72 | 10^-4^ | chromosome replication initiator |
| BSU07340 | *yfnA* | 0,72 | 10^-4^ | metabolite permease |
| BSU30350 | *yttB* | 0,73 | 10^-4^ | efflux transporter |
| BSU14630 | *speA* | 0,73 | 10^-4^ | arginine decarboxylase |
| BSU39960 | *yxaI* | 0,74 | 10^-4^ | hypothetical protein |
| BSU19160 | *yocC* | 0,78 | 10^-4^ | hypothetical protein |
| BSU23220 | *ypuG* | 0,79 | 10^-4^ | segregation and condensation protein A |
| BSU07430 | *yfmL* | 0,79 | 10^-4^ | ATP-dependent RNA helicase |
| BSU29660 | *rpsD* | 0,79 | 10^-4^ | 30S ribosomal protein S4 |
| BSU10160 | *yhgE* | 0,79 | 10^-4^ | methyl-accepting protein |
| BSU02870 | *yceA* | 0,80 | 10^-4^ | high affinity Zn(II) ABC transporter permease |
| BSU13130 | *proA* | 0,81 | 10^-4^ | gamma-glutamyl phosphate reductase |
| BSU11200 | *argJ* | 0,82 | 10^-5^ | ornithine acetyltransferase |
| BSU12100 | *yjeA* | 0,82 | 10^-4^ | secreted deoxyriboendonuclease |
| BSU23970 | *yqiY* | 0,84 | 10^-5^ | high affinity arginine ABC transporter permease |
| BSU29450 | *argG* | 0,84 | 10^-5^ | argininosuccinate synthase |
| BSU05470 | *ydfM* | 0,89 | 10^-4^ | divalent cation efflux transporter |
| BSU35250 | *ftsX* | 0,92 | 10^-4^ | cell-division ABC transporter |
| BSU23980 | *yqiX* | 0,95 | 10^-6^ | high affinity arginine ABC transporter binding lipoprotein |
| BSU11190 | *argC* | 0,97 | 10^-6^ | N-acetyl-gamma-glutamyl-phosphate reductase |
| BSU15490 | *pyrB* | 1,10 | 10^-7^ | aspartate carbamoyltransferase |
| BSU15480 | *pyrP* | 1,13 | 10^-6^ | uracil permease |
| BSU33330 | *yvsH* | 1,13 | 10^-6^ | lysine permease |
| BSU15470 | *pyrR* | 1,47 | 10^-7^ | bifunctional pyrimidine regulatory protein PyrR/uracil phosphoribosyltransferase |
